# Macrophage Cluster of Differentiation 163 promotes post‐infarction cardiac repair and preserves left ventricular function via osteopontin

**DOI:** 10.1002/ctm2.70662

**Published:** 2026-04-23

**Authors:** Jingyu Chen, Linjian Chen, Wei Huang, Gang Wang, Lin Wang, Wanchun Mei, Wei Ni, Yang Liu, Licheng Ding, Xiaofeng Ge, Zhaokai Li, Jing Yu, Shufen Huang, Jiayi Lin, Yifan Chen, Binni Cai, Peng Zhang, Cuilian Dai, Binbin Liu

**Affiliations:** ^1^ Xiamen Cardiovascular Hospital School of Medicine Xiamen University Xiamen China; ^2^ Department of Cardiology The Second Affiliated Hospital Chongqing Medical University Chongqing China; ^3^ Sir Run Run Shaw Hospital School of Medicine Zhejiang University Hangzhou China

**Keywords:** CD163, heart failure, myocardial infarction, osteopontin, ventricular dilation

## Abstract

**Background:**

Cardiac macrophages (cMacs) have been implicated in myocardial repair following myocardial infarction (MI), yet their therapeutic potential in ischaemic cardiomyopathy (ICM) remains limited by an incomplete understanding of their molecular regulation. Cluster of Differentiation 163 (CD163) is highly expressed in these macrophages, yet its functional role in regulating post‐MI cardiac repair remains unknown.

**Methods:**

A cross‐sectional clinical study was conducted to assess the association between circulating soluble CD163 concentration and heart failure due to ICM. To investigate the functional contribution of CD163 in ICM, wild‐type (WT) and *Cd163* ^−^ *^/^* ^−^ mice were subjected to permanent ligation of the left anterior descending coronary artery. Single‐cell RNA sequencing was employed to analyse transcriptional changes in cardiac immune cells. Recombinant osteopontin (OPN) and CD163 were administered to assess their therapeutic effect in *Cd163* ^−^ *^/^* ^−^ mice.

**Results:**

Circulating soluble CD163 levels were markedly elevated in patients with ICM‐induced heart failure compared with individuals without heart failure (median difference 34.5 ng/mL, IQR 13.6–54.6 ng/mL, *p* = .002) and showed a positive correlation with the extent of systolic dysfunction and left ventricular (LV) dilation. *Cd163* ^−^ *^/^* ^−^ mice displayed aggravated LV systolic dysfunction, reduced ejection fraction and fractional shortening, impaired myocardial strain and reduced relative wall thickness post‐MI. Recombinant CD163 protein could reverse systolic dysfunction and LV dilation in *Cd163* ^−^ *^/^* ^−^ mice after MI. CD163 was predominantly expressed in CCR2^−^ resident cMacs, and CD163 deficiency altered transcriptional programmes of macrophages without affecting their polarization status, with enrichment in cytokine signalling and extracellular matrix‐related pathways. *Spp1* (OPN) expression was significantly downregulated in *Cd163* ^−^ *^/^* ^−^ hearts under both sham and MI conditions. Administration of recombinant OPN improved systolic function, reduced ventricular dilation, decreased fibrotic scar size and restored the elastin‐to‐collagen ratio in *Cd163* ^−^ *^/^* ^−^ mice.

**Conclusions:**

In cMacs, CD163 contributes to post‐MI repair by upregulating OPN expression, which in turn helps maintain systolic function.

**Key points:**

- Soluble CD163 is elevated in ICM and correlates with LV dysfunction.
- *Cd163* ^−^ *^/^* ^−^ mice show worsened post‐MI systolic dysfunction and dilation.
- Recombinant CD163 reverses dysfunction in *Cd163* ^−^ *^/^* ^−^ mice.
- OPN restores function and improves LV structure in *Cd163* ^−^ *^/^* ^−^ mice.

## Graphical abstract

• Circulating soluble CD163 is elevated in ischaemic cardiomyopathy and correlates with systolic dysfunction severity and ventricular dilation.

• Macrophage CD163 protects against post‐infarction systolic dysfunction and left ventricular dilation in mice.

• Loss of CD163 decreases macrophage osteopontin expression.

• Local osteopontin administration restores systolic function, reduces scar area and rebalances the elastin‐to‐collagen ratio in CD163‐deficient hearts.

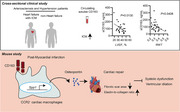

## 1 INTRODUCTION

Heart failure is the end‐stage manifestation of diverse cardiovascular disorders and is characterized by the inability of the heart to deliver adequate blood flow to satisfy the metabolic requirements of the body at rest or during exertion. It remains one of the major causes of hospitalization and death worldwide.^1^ Ischaemic cardiomyopathy (ICM) is a major aetiology of heart failure, characterized by significant left ventricular (LV) systolic dysfunction in the presence of severe obstructive epicardial coronary artery disease. Myocardial infarction (MI) is a primary cause of ICM, ultimately leading to heart failure, which encompasses cardiomyocyte necrosis followed by a complex process of cardiac repair and structural remodelling.^2^ Although myocardial revascularization and other pharmacological therapies have been advocated to improve myocardial function and clinical outcomes in ICM,^3^ MI is the most prevalent cause of heart failure and contributes significantly to its associated morbidity and mortality.^4^ The cellular mechanisms underlying these processes, however, remain incompletely understood.

Cardiac repair and healing are essential for the formation of connective tissue, which is critical for maintaining structural integrity and mechanical performance of the failing heart after MI.^5^ Myofibroblasts produce collagen and elastin, contributing to extracellular matrix (ECM) formation that supports cardiac repair. In mice, this reparative phase typically begins around Day 3–4 post‐MI and continues until approximately Day 14–28 post‐MI.^6^ Inadequate or insufficient repair can progressively lead to scar extended, adverse ventricular remodelling and ultimately heart failure. The loss of elastic recoil contributes to thinning and expansion of the infarct region, whereas elastin supplementation has been shown to improve cardiac systolic function.^7^, ^8^ Therefore, understanding the underlying regulatory mechanisms of myocardial repair is essential for the development of novel therapeutic strategies to prevent heart failure following MI.

Following MI, an immediate influx of CCR2^+^ macrophages and monocytes occurs in the heart, which progressively transition into reparative macrophages characterized by alternative (M2) polarization or CCR2^−^ resident populations to promote wound healing.^4^, ^9^ In mouse models, the depletion of M2 macrophages leads to ventricular dilation and impaired LV systolic function.^10^, ^11^ Cardiac macrophages (cMacs) modulate myocardial repair via cytokines and chemokines, such as IL‐1β, osteopontin (OPN) and TGF‐β, which play essential roles in myofibroblast activation and scar formation.^12^, ^13^ Although reparative cMacs are known to regulate cytokine and chemokine production and contribute to cardiac repair,^14^, ^15^ the detailed molecular mechanisms by which macrophages orchestrate cardiac repair and remodelling remain poorly understood.

Predominantly expressed by M2‐like macrophages, Cluster of Differentiation 163 (CD163) functions as a macrophage‐restricted scavenger receptor.^12^, ^16^ In humans, membrane‐bound CD163 can be cleaved at Arg‐Ser‐Ser‐Arg sites by A Disintegrin and Metalloproteinase 17 (ADAM17), releasing soluble CD163 (sCD163) into circulation.^17^ Clinical studies have shown that elevated circulating sCD163 levels predict an increased risk of both all‐cause and cardiovascular‐specific mortality, as well as incident heart failure.^18^ Furthermore, our recent work suggested that CD163 in macrophages contributes to the regulation of IL‐10 expression, thereby inhibiting heart failure triggered by pressure overload in mice.^19^ Thus, CD163 does not merely serve as a marker of macrophages but may also exert specific functions in macrophages. However, the precise molecular roles of CD163 in reparative macrophages during post‐MI cardiac repair remain largely unknown.

In this study, the association between CD163 and ICM was initially identified through a cross‐sectional clinical analysis. The dynamic changes in CD163 expression were subsequently examined in a mouse model following MI. To explore the functional contribution of CD163 to MI‐induced alterations of LV systolic function and structural remodelling, CD163‐deficient (*Cd163* ^−^ *^/^* ^−^) mice and recombinant mouse CD163 protein were employed. Single‐cell RNA sequencing (scRNA‐seq) was conducted to investigate how CD163 deficiency affects the transcriptional landscape of cMacs. OPN emerged as a key effector molecule regulated by CD163 in macrophages. To validate the contribution of OPN to LV dysfunction and impaired cardiac repair observed in *Cd163* ^−^ *^/^* ^−^ mice, exogenous OPN was administered in vivo. Therefore, this study aimed to investigate how CD163 in macrophages regulates cardiac repair and LV function following MI.

## 2 METHODS

### 2.1 Mice

Male mice (C57BL/6J) at 8 weeks old were used to minimize variability associated with hormonal cycles in females. *Cd163* ^−^ *^/^* ^−^ mice were backcrossed to the C57BL/6J genetic background for more than 10 generations and used alongside their wild‐type (WT) littermates as experimental controls.^19^, ^20^ The genotype was confirmed by PCR (Figure S1A). All animals were maintained under specific pathogen‐free conditions with a 12‐h light/dark cycle and ambient temperature controlled at 22–23°C at the animal facility of Xiamen University. For tissue collection, mice were deeply anaesthetized using 5% isoflurane delivered via inhalation, followed by euthanasia through exsanguination and heart excision. Hearts were perfused through the left ventricle with phosphate‐buffered saline (PBS) and 10% potassium chloride (KCl) before collection. All procedures were approved by the Xiamen University Animal Ethics Committee (Protocol No. XMULAC20200150) and adhered to the standards set by Directive 2010/63/EU of the European Parliament and the US National Institutes of Health guidelines on animal welfare.

### 2.2 MI surgery and mice treatment

MI was generated in male mice by permanently ligating the left anterior descending (LAD) coronary artery, following established protocols.^21^ One day prior to surgery, chest hair was removed with depilatory cream. Mice were anaesthetized with 2% isoflurane and placed on a heating pad to maintain normothermia. After disinfecting the skin with povidone‐iodine, a 1 cm left thoracic incision was made to expose the fourth intercostal space. The pleura and pericardium were opened, and the heart was gently exteriorized. The left coronary artery was ligated 1–2 mm below the left atrial appendage using a 6‐0 silk suture, and successful ligation was confirmed by pallor of the anterior LV wall. The heart was then returned to the thoracic cavity, residual air was evacuated, and the chest was closed. Mice typically recovered within 3–5 min under observation. Sham‐operated mice underwent the same procedure without ligation.

Recombinant mouse CD163 protein (ABclonal) was reconstituted in sterile PBS. The protein, purified from HEK293 cells with a purity exceeding 95%, was administered to mice at a dosage of  .05 mg/kg/day via intraperitoneal injection. Treatments were performed three times per week, starting from the day of surgery until 28 days post‐MI. An equivalent dose of mouse serum albumin (MSA) was administered to the control group following the same schedule.

Recombinant OPN was obtained from ABclonal. The protein, corresponding to amino acids Leu17 to Asn294 of mouse OPN and expressed in HEK293 cells, was purified to a purity of >95% and exhibits extensive post‐translational modifications, with an apparent molecular weight of 45–60 kDa. The protein was dissolved in sterile PBS at a concentration of 5 ng/µL. OPN or MSA was dissolved in sterile PBS. A total of 200 ng OPN (40 µL), MSA or an equal volume of PBS was injected at 2–3 sites along the infarct border zone immediately after LAD ligation using a Hamilton syringe with a 30‐ga, 4 mm needle.

### 2.3 Echocardiography

Transthoracic echocardiography was performed with a Vevo 2100 system (VisualSonics Inc.) and a 22–55 MHz MS550D transducer to evaluate cardiac structure and function.^19^ Under anaesthesia with 1.5%–2.0% isoflurane, two‐dimensional B‐mode and M‐mode images were obtained from the parasternal long‐axis view (PLAX) at the level of the papillary muscles. Data from echocardiographic images were processed and analysed using Vevo Lab software. All procedures were well tolerated, and mice recovered without complications. Investigators were blinded to group allocation during image acquisition and analysis. Relative wall thickness (RWT) was calculated as 2 × LV posterior wall thickness at end‐diastole (LVPW, d) divided by LV end‐diastolic diameter (LVEDD).

### 2.4 Histological analysis and immunohistochemistry (IHC)

Excised hearts were fixed in 4% paraformaldehyde at 4°C overnight, followed by paraffin embedding. Transverse sections (5 µm thick) were prepared for histological analysis. Collagen deposition was examined using Masson's trichrome staining, whereas elastin and collagen content were analysed via Verhoeff's elastin–Van Gieson (EVG) staining.^22^ IHC for CD163 was conducted to identify CD163‐positive cells in heart. Fiji was used to quantification.^23^ Antibodies and kits used are listed in Table S1. Scar thickness was assessed on Masson's trichrome–stained sections. In each infarcted region, eight evenly spaced points were selected along the ventricular wall, and the scar thickness was measured perpendicular to the wall at these sites. Relative scar thickness was normalized to the mean scar thickness of the WT‐MI group.

### 2.5 Flow cytometry

Whole hearts were minced and enzymatically digested in RPMI 1640 supplemented with 1% foetal bovine serum, .1 mg/mL Liberase and 1 µg/mL DNase I for 35 min at 37°C. After filtration, the resulting cell suspension underwent density gradient centrifugation using 40% and 70% Percoll to enrich immune cells. Prior to staining, cells were incubated with an Fc receptor blocking reagent in MACS buffer (PBS containing 1% BSA and 2 mM EDTA) for 10 min on ice to minimize nonspecific binding. Dead cells were excluded using a fixable viability dye, and surface markers were labelled with fluorophore‐conjugated antibodies during a 30‐min incubation on ice. After washing, samples were analysed on an LSR Fortessa flow cytometer (BD Biosciences, USA), and data were analysed using FlowJo software (version 10.0.7). The antibodies utilized are listed in Table S1.

### 2.6 2,3,5‐Triphenyltetrazolium chloride (TTC) staining

Mouse hearts were excised after PBS and 10% KCl perfusion, transversely sectioned into five slices and incubated in 2% freshly prepared 2,3,5‐triphenyltetrazolium chloride (TTC) solution at 37°C for 15 min in the dark with occasional shaking. Stained slices were briefly dried and photographed. The percentage of interact area in LV was quantified by Fiji.

### 2.7 scRNA sequencing

Whole hearts were enzymatically digested as described before, and CD45^+^ immune cells were isolated by fluorescence‐activated cell sorting (FACS) using a BD FACSAria III. Approximately 30 000 sorted cells were processed for single‐cell suspension preparation and library construction using a 5′ transcriptome library kit according to the manufacturer's protocol. Libraries were sequenced on the BGI T7 platform, generating 150‐bp paired‐end reads. Gene expression count matrices were produced using MobiVision v3.2 software.

Data processing was performed in R using the Seurat package. Cells were filtered using the following criteria: the number of detected genes (nFeature_RNA) between 400 and 4000, and mitochondrial gene percentage (percent.mt) below 10. Dimensionality reduction was conducted with a minimum distance (min.dist) of .02, and clustering was performed at a resolution of .08. T cells were further subclustered at a resolution of .5. Cell type annotation was carried out using the celldex packages *MouseRNAseqData* and *ImmGenData*. Monocytes and macrophages, T‐, B‐ and NK cells were subsetted for further clustering analysis. Differentially expressed genes (DEGs) between WT and *Cd163* ^−^ *^/^* ^−^ groups were identified using a minimum percentage expression (min.pct) of .25, with significance thresholds set at fold change >2 and adjusted *p*‐value < .05.

### 2.8 RNA sequencing

LV was dissected from the infarct zone (anterior wall) and remote zone (posterior wall) of each heart. One sample contains two to three mice. Total RNA was extracted using TRIzol reagent according to the manufacturer's protocol.^24^ Polyadenylated RNA was enriched and converted into cDNA libraries using a commercial library preparation kit. Libraries were multiplexed and sequenced to produce 150‐base pair paired‐end reads.

Sequencing reads were aligned to the mouse reference genome (GRCm39) with HISAT2 v2.2.1.^25^ Gene‐level counts were obtained using featureCounts (v2.0.1).^26^ Downstream statistical analysis was carried out in R (v4.2.1). Genes with low expression, which are defined as zero counts in more than 25% of samples, were excluded from analysis. Differential expression was assessed using the limma package, modelling gene counts with a generalized linear approach. Functional annotation and enrichment analysis were performed using the clusterProfiler package (v4.4.4).

### 2.9 Cross‐sectional study

Eligible individuals were aged 18 years or older, with a documented history of hypertension^27^ and a confirmed diagnosis of atherosclerosis. Participants were excluded if they refused or were unable to comply with study procedures or provide written informed consent. Patients with heart failure due to non‐ICM aetiologies were excluded, as were individuals with Stage 4 or 5 chronic kidney disease who were on long‐term haemodialysis or expected to require it within 6 months. Those requiring ventricular assist devices or listed for cardiac transplantation within the next 6 months were also excluded. Patients with an LV ejection fraction (LVEF) between 40% and 50%, elevated liver enzymes (aspartate aminotransferase or alanine aminotransferase levels exceeding three times the upper limit of normal), active cancer treated with chemotherapy or radiation within the past year, and individuals with autoimmune disorders or immunodeficiency were also excluded from the study. Based on LVEF and ICM aetiology, patients were categorized into two groups: a heart failure group with LVEF ≤ 40% and confirmed ICM, and a control group with preserved cardiac function (LVEF ≥ 50%) without heart failure. The study was conducted in at the Xiamen Cardiovascular Hospital affiliated with Xiamen University protocol with the approval from its Ethics Committee (2024YLK33). All participants gave written informed consent, and the study complied with the Declaration of *Helsinki*.^28^

Following an overnight fast of at least 12 h, peripheral blood was drawn into EDTA tubes and kept at 4°C until processing. Plasma was separated, aliquoted and stored at −80°C within 1 h. Plasma concentrations of sCD163 were measured using enzyme‐linked immunosorbent assay (ELISA). Prior to analysis, plasma samples were diluted at a 1:100 ratio. A commercially available ELISA kit for CD163, with a detection range from .156 to 5 ng/mL, was utilized for quantification.

To detect a 30% difference in serum sCD163 concentration, assuming a standard deviation (SD) of 55.5 ng/mL based on previous reports,^19^ with a two‐sided significance level (*α*) of .05, 80% power (1 − *β* = .8) and a 2:1 allocation ratio (control:experimental), the required sample sizes were estimated using the following formula for comparing two means with unequal group sizes: *n* = (*Z*_1−_ *_α_*_/2_ + *Z*_1−_ *_β_*)^2^ × (*σ* ^2^(1 + 1/*k*))/Δ^2^. The estimated sample sizes were 54 subjects for the control group and 27 subjects for the experimental group. Therefore, 60 patients were recruited for the control group and 30 for the experimental group.

### 2.10 Western blot

The infarct zone (anterior wall) of each heart was dissected. Samples were lysed in M‐PER lysis buffer with protease and phosphatase inhibitors. Proteins were quantified with a bicinchoninic acid assay, and equivalent amounts were subjected to SDS–PAGE followed by transfer onto PVDF membranes. Membranes were blocked with 3% non‐fat dry milk in TBS‐T (.1% Tween‐20) for 2 h at room temperature then incubated overnight at 4°C with primary antibodies diluted 1:2000 in 3% BSA in TBS‐T. Membranes were washed and incubated with HRP‐conjugated secondary antibodies for 2 h at room temperature, followed by the detection of protein bands via ECL on a Bio‐Rad platform. The Western blot bands were quantified by densitometric analysis using Fiji. Whole gel images are shown in Figure S11. Antibody details are shown in Table S1.

### 2.11 Statistics

Data analysis was conducted using Prism 9 and SPSS version 29.0. The specific statistical methods for each experiment are detailed in the relevant figure legends or tables. Prior to analysis, all datasets were evaluated for normal distribution and variance equality. Parametric tests, such as the *t*‐test for two‐group comparisons and ANOVA for multiple groups, were applied to data meeting these assumptions. When data deviated from normality, appropriate non‐parametric tests were utilized. In cases of unequal variances across groups, adjustments were made to the *p*‐values. To explore the relationship between sCD163 levels and heart failure incidence, binary logistic regression models were employed, both unadjusted and adjusted for potential confounders. The relationship between sCD163 levels and the continuous variables was analysed using simple linear regression. Results are expressed as mean ± SD or median with interquartile ranges (Q1–Q3). Statistical significance was defined as *p* < .05, with the following notation: ns (not significant, *p* > .05), ^*^ (*p* < .05), ^**^ (*p* < .01) and ^***^ (*p* < .001).

## 3 RESULTS

### 3.1 Higher sCD163 levels are associated with more severe LV dilation and systolic dysfunction in patients with ICM

**FIGURE 1:**
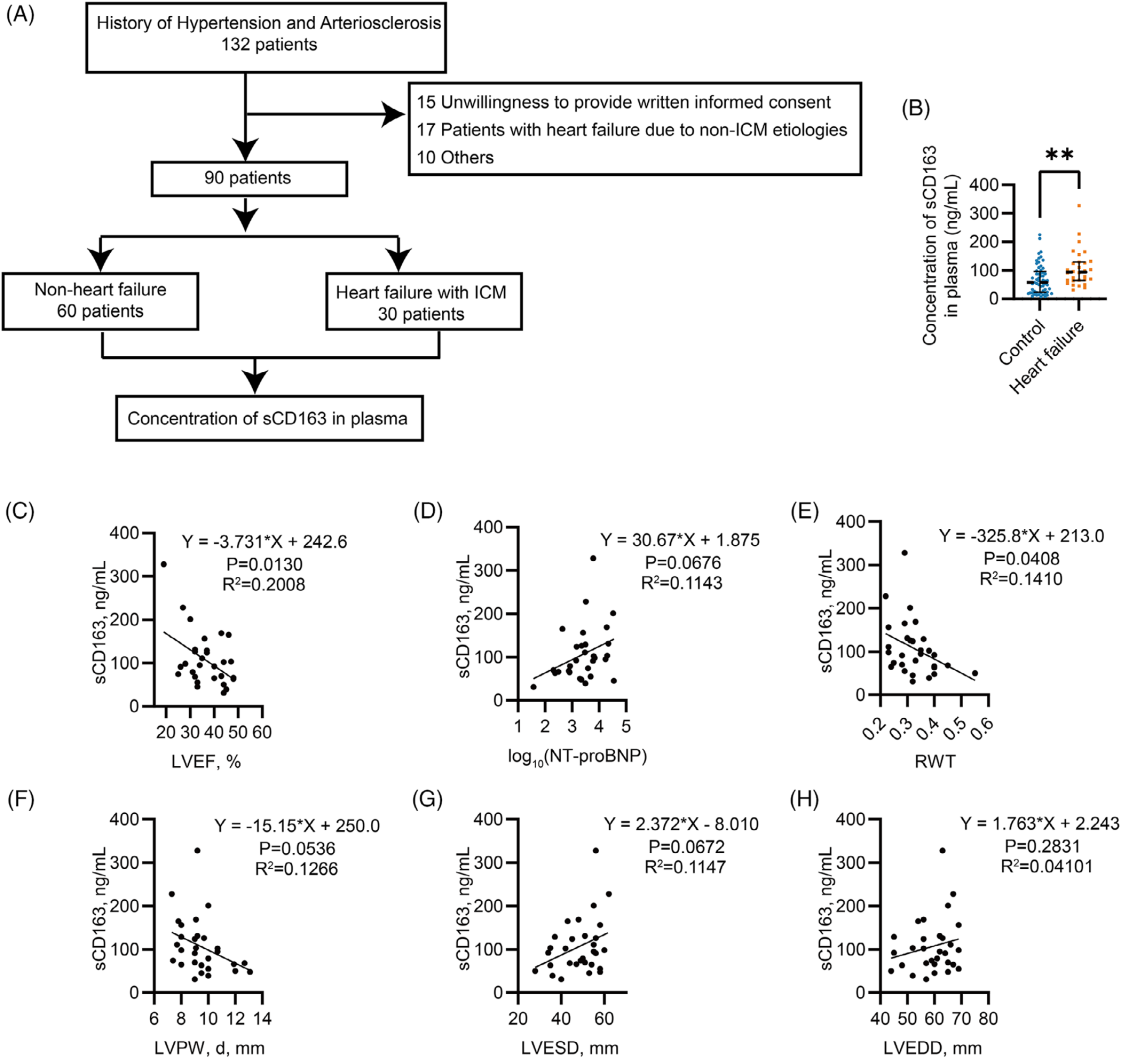
Higher soluble CD163 (sCD163) levels are associated with more severe left ventricular (LV) dilation and systolic dysfunction in patients with ischaemic cardiomyopathy (ICM). (A) Flowchart of patient enrolment. (B) Plasma concentrations of soluble Cluster of Differentiation 163 (CD163) (*n* = 60 and 30), analysed by Mann–Whitney test. Correlations between circulating sCD163 concentration and (C) LV ejection fraction (LVEF), (D) log_10_ of N‐terminal pro‐B‐type natriuretic peptide [log_10_(NT‐proBNP)], (E) relative wall thickness (RWT), (F) LV posterior wall thickness at diastole (LVPW, d), (G) LV end‐systolic diameter (LVESD) and (H) LV end‐diastolic diameter (LVEDD) are shown. Each data point represents an individual patient (*n* = 30). Statistical analysis was performed using simple linear regression. *p* values are indicated by asterisks: *p* < 0.05 (*), *p* < 0.01 (**), *p* < 0.001 (***).

To explore the clinical relationship between CD163 and ICM, we conducted a cross‐sectional study involving blood samples from patients with hypertension and atherosclerosis. A total of 132 individuals were initially screened from 15 July 2024 to 1 June 2025 at Xiamen Cardiovascular Hospital. Among them, 17 patients with heart failure unrelated to ICM and 25 patients with other exclusionary conditions were removed from the analysis (Figure 1A). Ultimately, the cohort was divided into two groups: 60 patients without heart failure and 30 patients diagnosed with heart failure secondary to ICM.

The baseline characteristics of these groups are summarized in Table S2. The median ages were 66.0 years (IQR 60.0–72.5) for the non‐heart failure group and 71.0 years (IQR 64.8–77.0) for the heart failure group. Male proportions were 60.0% and 66.7%, respectively. Body mass index was similar between groups (26.2 [24.0–28.6] vs. 24.7 [21.4–27.2]). No significant differences were found in smoking status, alcohol consumption, blood pressure or comorbidities, such as dyslipidaemia, atrial fibrillation and cerebrovascular accidents. However, the prevalence of diabetes was slightly higher in the heart failure group (*p* = .036). Laboratory results showed that patients with heart failure had significantly elevated N‐terminal pro B‐type natriuretic peptide (NT‐proBNP), and high‐sensitivity cardiac troponin T levels compared to controls. Echocardiographic evaluation revealed a significant reduction in LVEF in the heart failure group, along with increased left atrial diameter, LVEDD and LV end‐systolic diameter (LVESD), indicating notable ventricular dilation in patients with ICM‐related heart failure. Chronic kidney disease was more prevalent in the heart failure group, consistent with the elevated serum creatinine and decreased estimated glomerular filtration rate often seen as comorbidities in heart failure patients. Medication usage reflected typical heart failure management, with higher proportions of patients in the ICM group receiving loop diuretics, mineralocorticoid receptor antagonists, sodium–glucose cotransporter 2 (SGLT2) inhibitors and vericiguat. Together, these findings indicate that patients with ICM‐associated heart failure exhibit aggravated LV systolic dysfunction, ventricular dilation and related comorbidities, alongside standard heart failure pharmacotherapy.

For outcomes, the median plasma sCD163 concentration in the heart failure with ICM group at 93.8 ng/mL (IQR 64.4–129.3) was significantly increased compared to 58.2 ng/mL (IQR 23.2–96.6) in the non‐heart failure group, yielding a median difference of 34.5 ng/mL (95% CI 13.6–54.6, *p* = .002) (Figure 1B). Logistic regression analysis identified a positive correlation between sCD163 levels and heart failure risk (odds ratio [OR]: 1.012 per ng/mL increase; 95% CI: 1.003–1.021; *p* = .007). This association remained significant after adjusting for baseline demographic and clinical variables (Table 1), highlighting the clinical relevance of sCD163 in heart failure among patients with ICM.

**TABLE 1:** Outcomes.

	Control (*n* = 60)	Heart failure (*n* = 30)	
Median (Q1–Q3), ng/mL	58.2 (23.2–96.6)	93.8 (64.4–129.3)	*p*‐value
Median difference (95% CI), ng/mL	34.5 (13.6–54.6)		.002^**^
Model 1			
Odds ratios (95% CI)	1.012 (1.003–1.021)		.007^**^
Model 2			
Odds ratios (95% CI)	1.013 (1.002–1.024)		.021^*^
Model 3			
Odds ratios (95% CI)	1.011 (1.001–1.002)		.034^*^

*Note*: Model 1: Binary logistic regression without adjusting for confounding factors. Model 2: Binary logistic regression adjusted for systolic blood pressure, history of diabetes mellitus and triglyceride levels. Model 3: Binary logistic regression adjusted for age, sex, body mass index, smoking status, alcohol consumption, total cholesterol, high‐density lipoprotein cholesterol and low‐density lipoprotein concentration. *p*‐values are denoted as follows:

* *p* < .05,

** *p* < .01. *p*‐values were calculated using the Mann–Whitney test.

Further, the association between circulating sCD163 and LV function as well as LV dilation in patients with ICM was investigated. Circulating sCD163 concentration was negatively correlated with LVEF (*p* = .01) and showed a trend towards a negative correlation with NT‐proBNP, indicating that higher sCD163 levels are associated with more severe systolic dysfunction (Figure 1C,D). Furthermore, sCD163 levels were significantly negatively correlated with RWT^29^ (*p* = .04) (Figure 1E). Trends towards negative associations were also observed between sCD163 and LVPW, d (Figure 1F), whereas trends towards positive associations were observed with LVESD and LVEDD (Figure 1G,H), suggesting that higher sCD163 levels are associated with greater LV dilation in patients with ICM.

### 3.2 CD163 expression was decreased in the infarct zone during the reparative phase after MI

**FIGURE 2:**
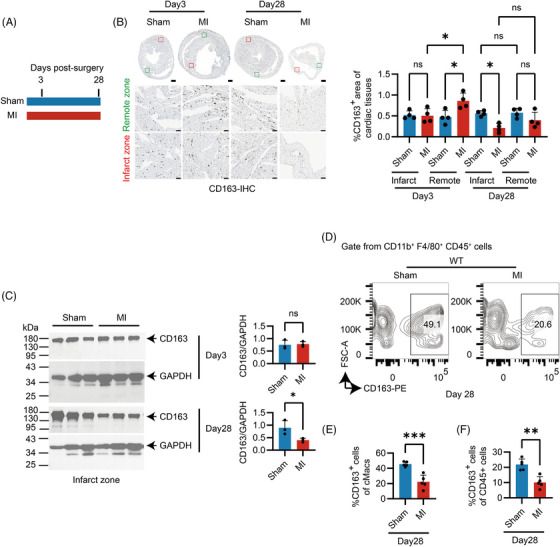
Cluster of Differentiation 163 (CD163) expression was decreased in the infarct zone during the reparative phase after myocardial infarction (MI). (A) Schematic diagram of the mouse study design. (B) Immunohistochemistry staining of CD163 in heart sections at Day 3 and 28 post‐MI. Scale bars, 500 and 50 µm. Boxed areas indicate the regions shown in the zoomed‐in images. Data points represent individual mice (*n* = 4) from two independent experiments and analysed by one‐way ANOVA with Tukey's multiple comparisons test. (C) Protein levels of CD163 expression in the heart at Day 3 and 28 post‐MI. Quantification of the CD163/GAPDH ratio is presented (*n* = 3). (D–F) Flow cytometric analysis of CD163^+^ cardiac macrophages (cMacs), defined as CD11b^+^F4/80^+^CD45^+^ cells, including representative plots (D), percentage of CD163^+^ cMacs among total cMacs (E) and percentage of CD163^+^ cMacs among total CD45^+^ cells (F). Each dot represents one mouse (*n* = 5), with data pooled from two independent experiments. Statistical analysis was performed using unpaired *t*‐test. See also Figure S1. WT, wild‐type. *p* values are indicated by asterisks: *p* < 0.05 (*), *p* < 0.01 (**), *p* < 0.001 (***).

To investigate how CD163 expression evolves after MI, mice underwent either sham operation or LAD ligation. CD163 expression was assessed at Day 3, corresponding to the end of the inflammatory stage in cardiac repair, and at Day 28, corresponding to the end of reparative phase following MI (Figure 2A). IHC analysis revealed that in the infarct zone, total CD163^+^ cell number remained unchanged at Day 3 but was significantly decreased by Day 28. In the remote zone, CD163^+^ signals significantly increased at Day 3 before declining at Day 28, indicating a spatial and temporal variation in CD163 expression following MI (Figure 2B and Figure S1B). Western blot analysis supported these findings, showing a consistent trend in CD163 protein levels in infarct zone over time (Figure 2C). Additionally, flow cytometry showed that the percentage of cMacs within CD45^+^ cells did not differ significantly between sham and MI‐treated groups in both WT mice (Figure S1D,E) However, in WT mice following MI, the proportion of CD163^+^ cMacs decreased significantly, from 47.8% to 24.0% within the cMac population and from 22.9% to 10.9% among CD45^+^ cells (Figure 2D–F). It is noteworthy that although CD163^+^ cells were reduced in number compared to the sham group, substantial CD163 expression remains detectable in both the infarct and remote zones during the reparative phase after MI. Due to significant differences in the immune microenvironment between sham and MI conditions, the functional implications of this residual CD163 expression need to clarify.

### 3.3 Macrophage CD163 protected against MI‐induced systolic dysfunction and LV dilation

**FIGURE 3:**
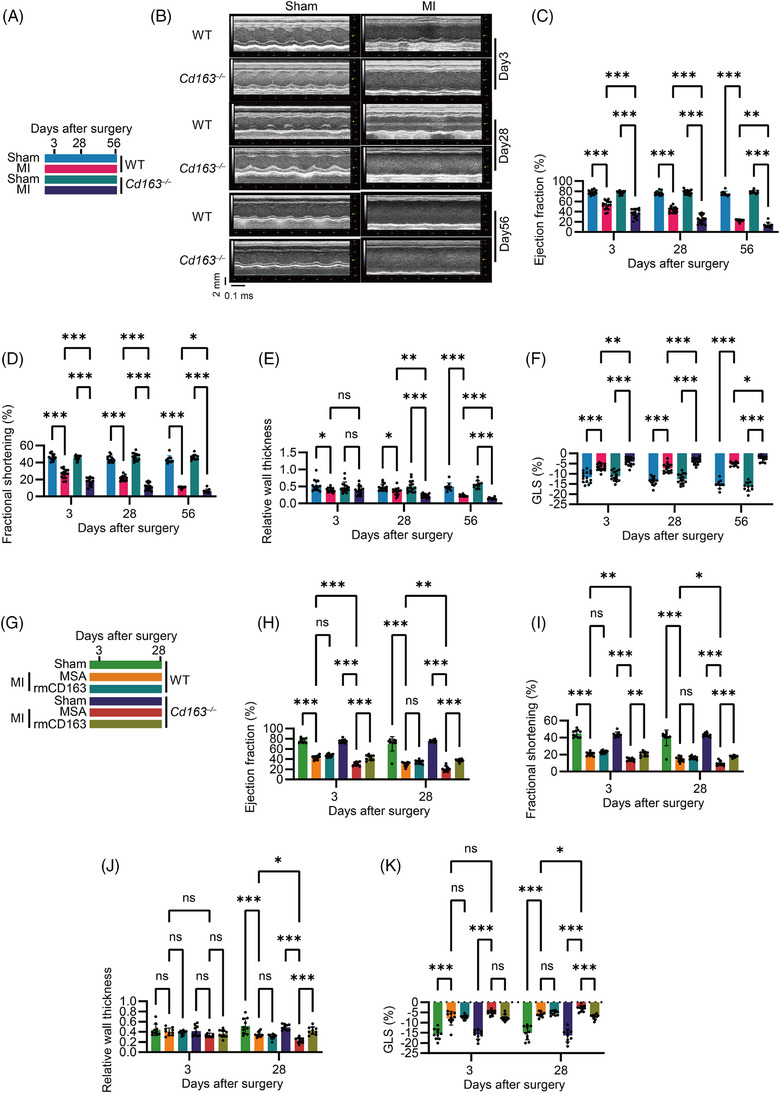
Macrophage Cluster of Differentiation 163 (CD163) protected against myocardial infarction (MI)‐induced systolic dysfunction and left ventricular (LV) dilation. (A) Schematic diagram of the mouse study design by using wild‐type (WT) and *Cd163* ^−^ *^/^* ^−^ mice subjected to left anterior descending (LAD) ligation or sham surgery. (B) Representative M‐mode echocardiography images analysed by parasternal long‐axis view (PLAX). Quantification of (C) ejection fraction, (D) fractional shortening, (E) relative wall thickness and (F) global longitudinal strain (GLS). Each dot represents one mouse (*n* = 15 for Days 3 and 28; *n* = 10 for Day 56) pooled from two to three independent experiments. (G) Schematic diagram of the study design using sham‐ or MI‐treated WT and *Cd163* ^−^ *^/^* ^−^ mice receiving recombinant mouse CD163 protein (.05 mg/kg/day) or mouse serum albumin (MSA) via intraperitoneal injection three times per week. Quantification of (H) ejection fraction, (I) fractional shortening, (J) relative wall thickness and (K) GLS. Each dot represents one mouse (*n* = 10) pooled from two independent experiments. Statistical analysis was performed using two‐way ANOVA followed by Tukey's multiple comparisons test. See also Figures S2–S4. *p* values are indicated by asterisks: *p* < 0.05 (*), *p* < 0.01 (**), *p* < 0.001 (***).

To investigate the functional role of CD163 in MI‐induced systolic dysfunction and ventricular remodelling, *Cd163* ^−/−^ and WT mice underwent either sham or MI surgery (Figure 3A). First, IHC suggested that CD163 expression was not detected in cardiomyocytes but was observed in non‐cardiomyocytes in WT mice. Further, flow cytometry confirmed that CD163 is expressed in cMacs, whereas no expression was observed in *Cd163* ^−/−^ mice. This finding is consistent with the previous understanding that CD163 is highly and exclusively expressed by cells of the monocytic–macrophage lineage.^30^ Thus, its genetic deletion primarily disrupts the expression of CD163 in macrophages and monocytes in heart (Figure 2B–F and Figure S1B,F).

Post‐MI, heart weight increased in both WT and *Cd163* ^−/−^ mice, with a significant increase observed at Day 28; however, no differences were detected between MI‐treated WT and *Cd163* ^−/−^ mice (Figure S2A,B). TTC staining at Day 3 and 28 post‐MI showed that the infarct area was enlarged following MI, but no differences were observed between the two genotypes, suggesting that CD163 plays a minor role in MI‐induced cardiomyocyte loss (Figure S2C,D). Echocardiography in the PLAX view was performed to assess LV structure and function at comparable heart rates (Figure 3B and Figure S3A,B). MI significantly reduced LVEF (Figure 3C) and fractional shortening (Figure 3D), with CD163 deficiency further exacerbating these impairments at Day 3, 28 and 56 post‐MI. MI‐induced reductions in RWT were also more pronounced in *Cd163* ^−/−^ mice at Day 28 and 56 (Figure 2G). Additionally, LVEDD and LVESD were increased in *Cd163* ^−/−^ mice compared to WT post‐MI (Figure S3C,D). Both LVPW and LV anterior wall thickness at end‐diastole and systole decreased after MI, CD163 deficiency induced a further modest reduction (Figure S3E–H). Global longitudinal strain (GLS), which is speckle‐tracking echocardiography parameters that assess myocardial contractile function,^31^ was measured at B‐mode at comparable heart rates (Figure S3I,J). MI‐induced reductions in GLS were further significantly exacerbated in *Cd163* ^−/−^ mice compared to WT mice (Figure 3F).

Cryo‐electron microscopy analysis revealed that the extracellular domain of CD163 forms multimers that mediate haemoglobin–haptoglobin endocytosis by macrophages.^32^, ^33^ Furthermore, as our previous study demonstrated that recombinant mouse CD163 can modulate cytokine expression in vitro,^16^ we administered recombinant CD163 protein to mice to investigate its direct functional role in the post‐MI (Figure 3G). CD163 protein significantly improved the reduced LVEF and fractional shortening in *Cd163* ^−/−^ mice, but not in WT mice at Day 3 and 28 post‐MI (Figure 3H,I, and Figure S4A–C). In addition, the reduced RWT in *Cd163* ^−/−^ mice was significantly restored at Day 28 post‐MI (Figure 3J). Consistently, LV diameter and LV wall thickness also showed corresponding improvements in *Cd163* ^−/−^ mice following CD163 protein administration (Figure S4D–I). Furthermore, a significant improvement in GLS was observed in *Cd163* ^−/−^ mice at Day 28 post‐MI after CD163 protein treatment (Figure 3K and Figure S4J,K). These findings suggest that the CD163 molecule in macrophages plays a protective role against MI‐induced systolic dysfunction and LV dilation.

### 3.4 CD163 deficiency suppressed OPN expression in cMacs following MI

**FIGURE 4:**
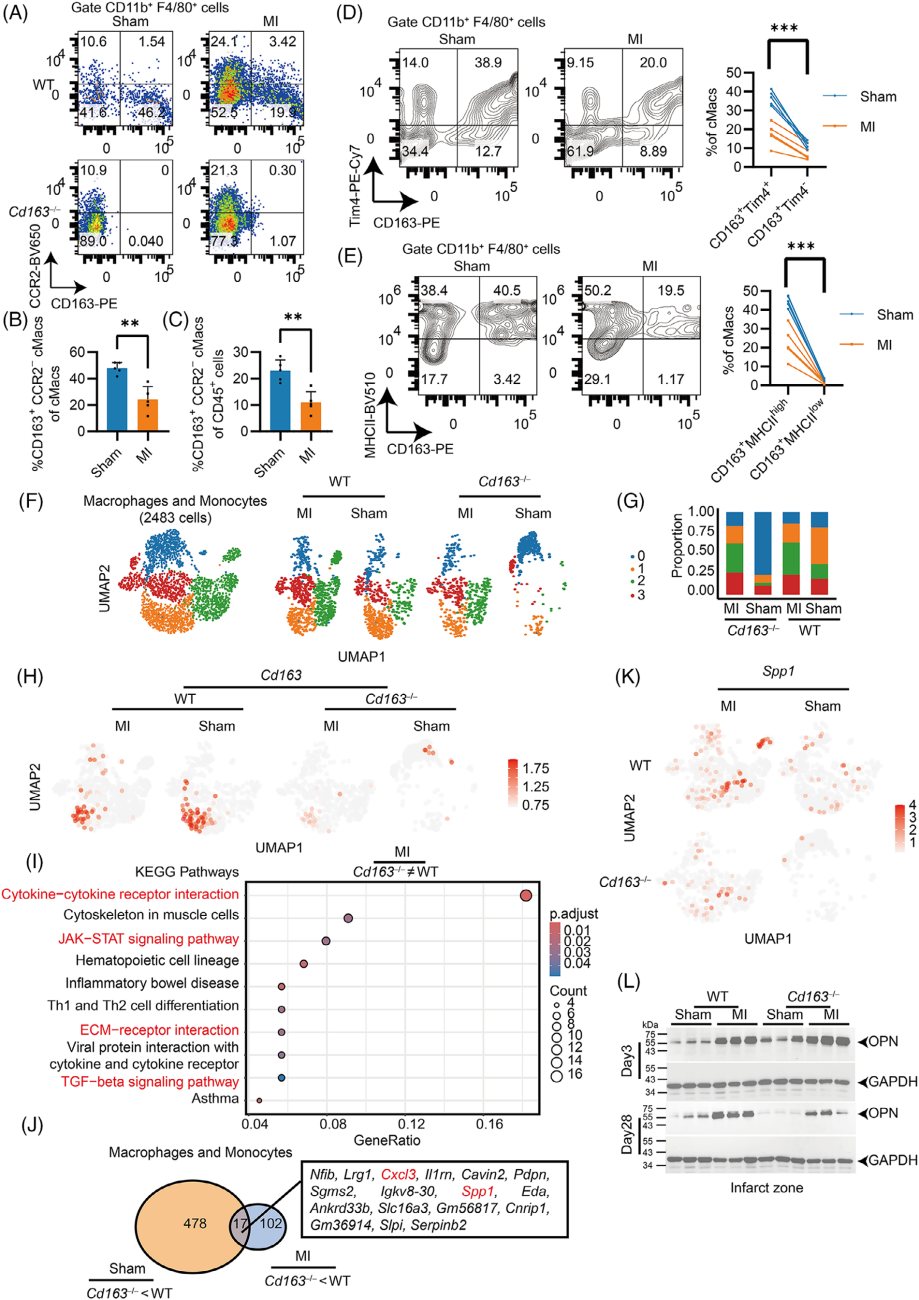
Cluster of Differentiation 163 (CD163) deficiency represses the expression of osteopontin (OPN) in cardiac macrophages (cMacs) during post‐myocardial infarction (MI) cardiac repair. (A) Representative flow cytometry plots showing the distribution of CD163 and CCR2 in cMacs. Percentages of CD163^+^CCR2^−^ cMacs among (B) total cMacs and (C) CD45^+^ cells. Data points represent individual mice (*n* = 5) from two independent experiments and statistics by unpaired *t*‐test. (D) Distribution of Tim4 and CD163 in cMacs and (E) distribution of MHC‐II and CD163 in cMacs. Data points represent individual mice (*n* = 10) from two independent experiments and statistics by paired *t*‐test. (F) CD45^+^ cells sorted at Day 28 post‐surgery for single‐cell RNA sequencing (scRNA‐seq), with 2483 macrophages and monocytes further subclustered. (G) Proportion of indicated clusters in each group. (H) Distribution of *Cd163* in the indicated groups. (I) KEGG pathway analysis of differentially expressed genes between wild‐type (WT) and *Cd163* ^−^ *^/^* ^−^ mice in the MI group. (J) Venn diagram showing genes significantly downregulated in *Cd163* ^−^ *^/^* ^−^ mice compared to WT in both sham and MI groups. (K) Distribution of *Spp1* in the indicated groups. (L) Protein levels of OPN in the indicated groups. Data represent three biological replicates per group. One representative out of three similar experiments is displayed. See also Figures S5–S8. *p* values are indicated by asterisks: *p* < 0.05 (*), *p* < 0.01 (**), *p* < 0.001 (***).

As CCR2^−^ or M2 macrophages have been reported to participate in cardiac repair after MI,^9^ we first sought to examine the distribution of CD163 in the cMacs subsets by flow cytometry. Although the percentage of CCR2^+^ macrophages among cMacs was observed to increase, nearly all CD163^+^ macrophages were CCR2^−^ under both Sham and MI conditions, with a significant decrease following MI (Figure 4A–C). Tim4 has been reported as a marker of resident macrophages,^34^ and the proportion of CD163^+^ Tim4^+^ cells was significantly higher than that of CD163^+^ Tim4− cells (Figure 4D). Additionally, almost all CD163^+^ macrophages expressed MHC‐II on their surface (Figure 4E). Taken together, these data suggest that CD163 is mainly expressed in resident CCR2^−^ macrophages under physiological conditions and during cardiac repair post‐MI.

To investigate the functional role of CD163 on macrophages after MI, particularly during the cardiac repair post‐MI, scRNA‐seq was performed on CD45^+^ cells isolated from the hearts of sham‐ and MI‐treated WT and *Cd163* ^−/−^ mice at Day 28 (Figure S5A). A total of 9335, 9450, 7144 and 8776 CD45^+^ cells were filtered, retained for clustering analysis and annotated from sham‐treated WT mice, MI‐treated WT mice, sham‐treated *Cd163* ^−/−^ mice and MI‐treated *Cd163* ^−/−^ mice, respectively (Figure S5B,C). Macrophages and monocytes were consistently identified in clusters characterized by a high expression of *Lyz2* and *Cd86* (Figure S5D). Notably, *Cd163* was specifically and abundantly expressed within the macrophage and monocyte clusters of WT mice, but absent in *Cd163* ^−/−^ mice (Figure S5D), confirming the successful clustering of macrophages and monocytes and validating the genotype‐specific expression pattern. A total of 2483 macrophages and monocytes were further subclustered (Figure 4F). In the sham group, Clusters 0 and 1 were predominant, whereas Clusters 2 and 3 were enriched in MI‐treated mice (Figure 4G). CD163^+^ cells were primarily localized in Cluster 1 and were nearly absent in Cluster 2 (Figure 3H). We next examined macrophage polarization markers. Classical M1 markers (CD80, TNF‐α) and M2 markers (CD206, CD301) did not show significant differences between WT and *Cd163* ^−/−^ mice after MI (Figure S6A), suggesting that CD163 deficiency did not markedly alter conventional M1/M2 polarization profiles. Compared to WT controls, sham‐treated *Cd163* ^−/−^ mice exhibited a marked increase in Cluster 0 alongside a concomitant decrease in Cluster 1. Functional enrichment revealed that Cluster 1 is enriched with genes involved in antigen presentation (*H2‐Aa*, *H2‐Ab1* and *Cd86*) and phagocytosis (*Cd163*, *Mertk* and *Msr1*). Conversely, Cluster 0 is characterized by a high expression of effector cytokines and cytotoxic molecules, including *Gzma*, *Gzmc*, *Ifng*, *Il2*, *Il4*, *Il5* and *Il17a*, indicating that macrophage CD163 also plays a pivotal role in maintaining immunological balance under steady‐state conditions (Figure S6B,C). DEGs between MI‐treated WT and *Cd163* ^−/−^ mice in macrophages and monocytes revealed that the altered genes were enriched in cytokine, JAK‐STAT signalling pathway and ECM‐related pathways (Figure 4I and Figure S6D). This led to the hypothesis that macrophage CD163 may influence ECM remodelling via specific cytokines. To explore this, we identified overlapping genes altered by CD163 deficiency under both sham and MI conditions. Among the downregulated genes, 17 were identified, including two cytokines that are *Spp1* (encoding OPN) and *Cxcl3* (Figure 4J). In contrast, *Ifng* and *Il5* were among in the 27 upregulated genes (Figure S6E). *Spp1* drew our attention because previous studies have shown that mice lacking OPN exhibit exacerbated ventricular dilation and impaired LV function following MI,^35^ and the relationship between OPN and CD163 in macrophages has not yet understood. Although the distribution of *Spp1* ^+^ and *Cd163* ^+^ cells in cMacs did not completely overlap (Figure 4K), a substantial portion of the CD163^+^ cells in MI‐treated WT mice coincided with *Spp1* expression. Western blot analysis further confirmed that OPN protein levels were reduced in both the infarct zones of *Cd163* ^−/−^ hearts compared to WT controls in Sham and MI mice at Day 28 post‐MI but not Day 3 post‐MI (Figure 4L). STAT3 phosphorylation was detected at Day 28 post‐MI in WT mice but was absent in *Cd163* ^−/−^ mice (Figure S6F). These findings indicate that CD163 is critical for OPN expression in cMacs during the reparative phase following MI.

Besides, to obtain a more comprehensive picture of the immune landscape regulated by CD163 during the reparative phase, we further analysed other immune cell subsets. Among T cells, both CD4^+^ and CD8^+^ T‐cell populations were examined. Based on the expression of CD44 and CCR7, these cells were further subclustered.^36^ We found that CCR7^low^ CD44^+^ effector memory T cells were increased after MI. Importantly, CCR7^high^ CD44^low^ central memory T cells were also increased in WT mice after MI, and their proportion was higher than that in *Cd163* ^−/−^ hearts (Figure S7A–C). This subset highly expressed *Cxcl1, Mmp2, Mmp9, Gzmb, Col1a1 and Spp1*, suggesting a potential contribution to cardiac remodelling and repair (Figure S7D). Interestingly, *Spp1* expression was also reduced in T cells from *Cd163* ^−/−^ mice in both sham and MI‐treated mice (Figure S7E). Furthermore, the analysis of B‐cell and NK‐cell subsets suggested that MI induced the activation of both B cells and NK cells, whereas CD163 deficiency did not markedly alter these subsets (Figure S8A–E). These results suggest that macrophage CD163 may influence T‐cell subset composition, which could be important for cardiac repair, with OPN serving as an important mediator of this process.

### 3.5 OPN administration alleviated systolic dysfunction and ventricular dilation in CD163‐deficient mice after MI

**FIGURE 5:**
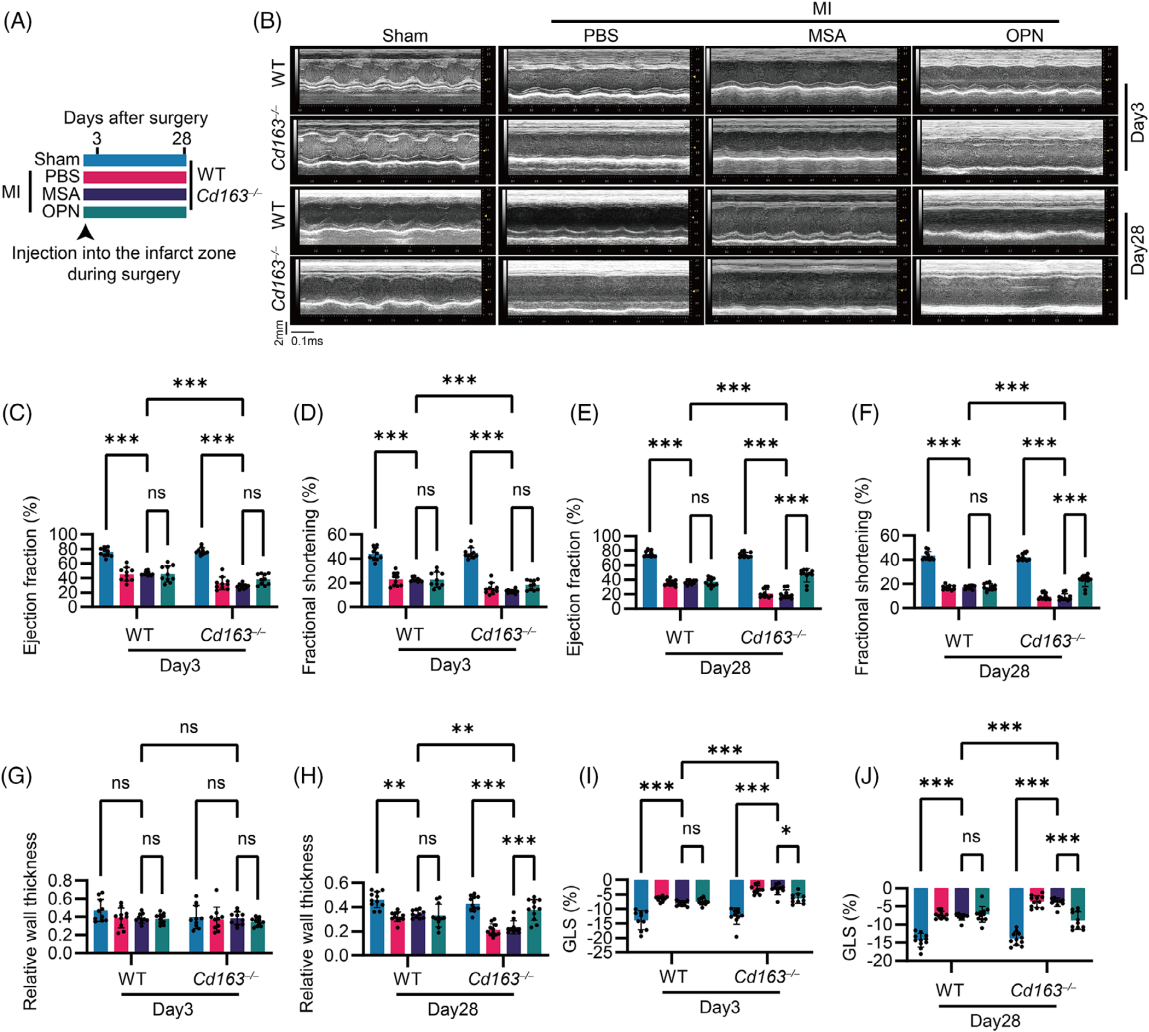
Osteopontin (OPN) administration alleviated systolic dysfunction and ventricular dilation in Cluster of Differentiation 163 (CD163)‐deficient mice after myocardial infarction (MI). (A) Schematic diagram of the mouse study design. Mice received 200 ng OPN, mouse serum albumin (MSA) or an equal volume of phosphate‐buffered saline (PBS) injected directly into the infarct zone during surgery. (B) Representative M‐mode echocardiography images. (C–J) Quantification of ejection fraction, fractional shortening, relative wall thickness and global longitudinal strain (GLS) at Day 3 and 28 post‐MI. Data points represent individual mice (*n* = 11 or 10 for MSA‐treatment) from two independent experiments. Statistical analysis was performed using two‐way ANOVA with Tukey's multiple comparisons test. See also Figure S9. WT, wild‐type. *p* values are indicated by asterisks: *p* < 0.05 (*), *p* < 0.01 (**), *p* < 0.001 (***).

To determine whether OPN contributes to the exacerbation of systolic dysfunction and ventricular dilation observed in CD163‐deficient mice after MI, we administered 200 ng of OPN directly into the infarct zone at the time of surgery,^37^ with equal volumes of PBS and MSA serving as negative controls (Figure 5A). Echocardiography in the PLAX view was performed to assess LV structure and function at comparable heart rates (Figure 5B and Figure S9A–C). In *Cd163* ^−/−^ mice, OPN administration significantly reversed the reductions in LVEF, fractional shortening and RWT at Day 28 post‐MI, but not at Day 3, whereas OPN had no significant effect on these parameters in WT mice (Figure 5C–H). LVEDD and LVESD were also reduced in MI‐treated *Cd163* ^−/−^ mice after OPN treatment only at Day 28 not Day 3 (Figure S9D–G). Similarly, GLS, measured at comparable heart rates, were improved by OPN in *Cd163* ^−/−^ mice, but not in WT mice post‐MI (Figure 5I,J and Figure S9H–K). Collectively, these findings indicate that OPN treatment mitigates systolic dysfunction and ventricular dilation in CD163‐deficient mice following MI, especially during the post‐infarction reparative phase.

### 3.6 OPN administration improved cardiac repair in CD163‐deficient mice following MI

**FIGURE 6:**
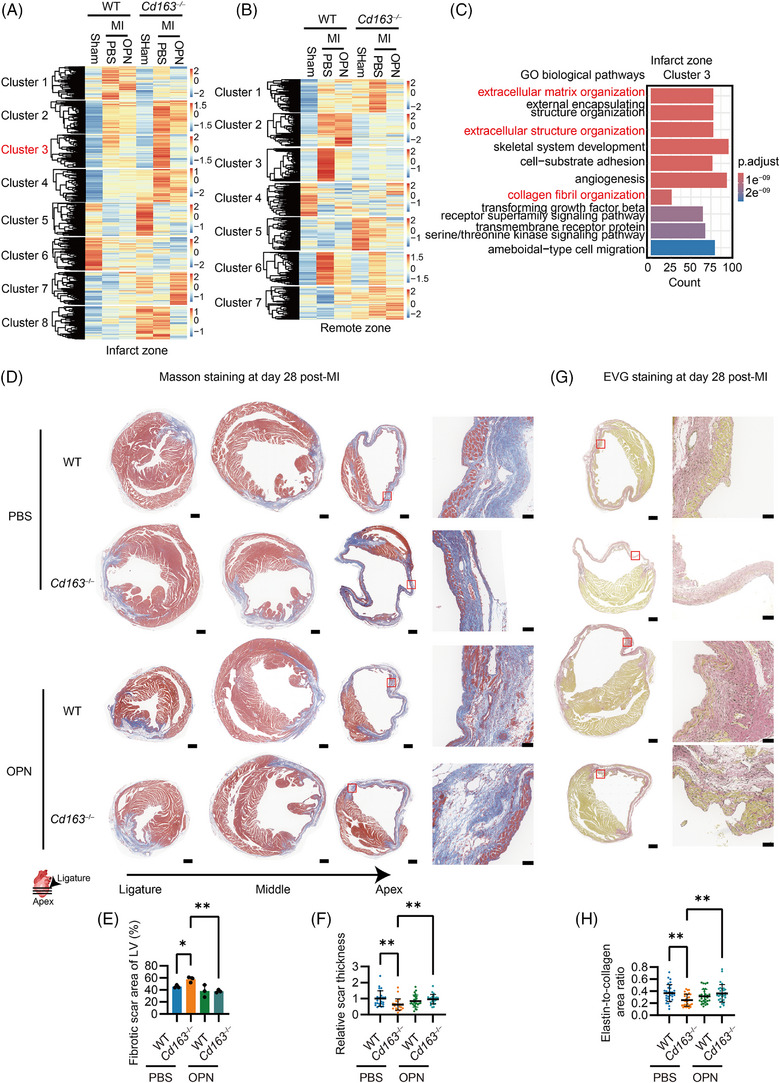
Osteopontin (OPN) administration improved cardiac repair in Cluster of Differentiation 163 (CD163)‐deficient mice following myocardial infarction (MI). (A, B, G) RNA‐seq of infarct and remote zones at Day 28 post‐MI. Mice received 200 ng OPN or phosphate‐buffered saline (PBS) injected into the infarct zone during MI or underwent sham surgery (wild‐type [WT] and *Cd163* ^−^ *^/^* ^−^). (A) Heatmap showing the average expression of differentially expressed genes (DEGs) in the infarct zone of PBS‐treated *Cd163* ^−^ *^/^* ^−^ MI mice (cut‐off: *p* < .05). Sample sizes: *n* = 3, 3, 3, 3, 2, 2, with each sample pooled from two to three mice. (B) Heatmap showing the average expression of DEGs in the remote zone compared with PBS‐treated *Cd163* ^−^ *^/^* ^−^ MI mice (cut‐off: *p* < .05); *n* = 3, 3, 3, 3, 3, 2, with each sample pooled from two to three mice. (C) GO biological pathway enrichment analysis of Cluster 3 genes identified in (A). (D) Masson's trichrome staining of heart sections from the indicated mice at Day 28 post‐surgery. Scale bar: 500 and 50 µm. Boxed areas indicate the regions shown in the zoomed‐in images. (E) Percentage of fibrotic scar area in the left ventricle. Data points represent individual mice (*n* = 3) from two independent experiments. Statistical analysis was performed using one‐way ANOVA with Holm‐Šídák's multiple comparisons test. (F) Relative scar thickness was normalized to the mean scar thickness of PBS‐treated WT mice following MI. Each data point represents an individual region (*n* = 24) collected from three mice across two independent experiments. Statistical significance was assessed using the Kruskal–Wallis test with Dunn's multiple comparisons. (G) Verhoeff's elastin–Van Gieson (EVG) staining showing elastin (black) and collagen (red) in heart sections from the indicated mice at Day 28 post‐surgery. Scale bar: 500 and 50 µm. Boxed areas indicate the regions shown in the zoomed‐in images. (H) Quantification of the elastin‐to‐collagen area ratio after excluding nuclear areas. Data points represent individual fields of view (*n* = 30) from three mice across two independent experiments. Statistical analysis was performed using the Kruskal–Wallis test followed by Dunn's multiple comparisons. See also Figure S10. *p* values are indicated by asterisks: *p* < 0.05 (*), *p* < 0.01 (**), *p* < 0.001 (***).

To investigate the effects of CD163 and OPN on cardiac repair, transcriptomic analysis was conducted on both the infarct and remote zones. Principal component analysis (PCA) revealed that the transcriptomic profiles of OPN‐treated WT and *Cd163* ^−/−^ mice in the infarct zone were more closely clustered compared to those treated with PBS, indicating that OPN treatment reduced the transcriptional differences caused by CD163 deficiency. This convergence was not observed in the remote zone (Figure S10A,B). DEGs between MI‐treated *Cd163* ^−/−^ heart and other groups were identified and clustered based on their expression profiles (Figure 6A,B). In the infarct zones, a total of eight gene expression clusters were identified. Notably, Cluster 3 consisted of genes that were upregulated in the hearts of MI‐treated *Cd163* ^−/−^ mice compared to WT controls. Importantly, OPN administration markedly suppressed the expression of these genes in *Cd163* ^−/−^ hearts relative to PBS treatment (Figure 6A). Functional enrichment analysis revealed that these genes were primarily associated with cytokine signalling and ECM‐related pathways (Figure 6C). In contrast, analysis of the remote zones revealed that Cluster 1 exhibited a potential trend in which OPN partially reversed the upregulation of genes caused by CD163 deficiency following MI (Figure 6B). However, these genes were mainly involved in translational processes (Figure S10C). Genes in Clusters 3 and 6, which were upregulated after MI, were suppressed by both CD163 deficiency and OPN treatment in the remote zone (Figure 6B). Functional enrichment analysis revealed that these genes were primarily associated with T cell proliferation and ECM organization (Figure S10D,E). The results suggest that OPN can reverse CD163 deficiency‐induced alterations in transcriptional profiles associated with ECM‐related pathways in the infarct zone.

Further, Masson's trichrome staining of heart sections revealed that CD163‐deficient mice exhibited impaired cardiac repair, as evidenced by a significant increase in fibrotic scar area percentage in the LV and reduced scar thickness. Notably, OPN supplementation markedly reversed these changes (Figure 6D–F and Figure S10F,G). Moreover, EVG staining was performed to evaluate the scar composition post‐MI. In *Cd163* ^−/−^ mice, the elastin‐to‐collagen ratio was significantly reduced, and this change was largely reversed by OPN treatment (Figure 6G,H and Figure S10H), suggesting that OPN mitigates CD163 deficiency–induced impairment in cardiac repair after MI.

## 4 DISCUSSION

Reparative macrophages, typically characterized as M2 and CCR2^−^ resident macrophages, promote post‐infarction cardiac repair.^9^, ^11^ Enhancing their polarization further supports cardiac healing preserves LV function and reduces ventricular dilation.^14^ CD163, a well‐recognized M2 macrophage marker.^16^, ^20^ Here, we found that elevated circulating sCD163 levels are associated with an increased risk of heart failure in patients with ICM. During the progression of HBV‐related acute‐on‐chronic liver failure, pro‐inflammatory monocytes were increased at the early stage, whereas blood CD163^+^ monocytes served as significant markers of disease deterioration, indicating that CD163 may play an important role in inflammation‐associated diseases, including heart failure and liver failure.^38^ In mice post‐MI, CD163 expression in the CCR2^−^ resident macrophages protected against systolic dysfunction and LV dilation, reduced fibrotic scar formation and increased the elastin‐to‐collagen ratio within the scar. These effects were mediated through the regulation of OPN expression, rather than by altering cMac polarization. Collectively, these results highlight that CD163 expression in reparative macrophages is essential for cardiac repair and preservation of LV function post‐MI, rather than serving merely as a macrophage marker. Beyond regulating macrophage polarization, targeting macrophage‐intrinsic molecules also represents a potential strategy to modulate the function of reparative macrophages.

Circulating sCD163 is generated by proteolytic cleavage of membrane‐bound CD163 on macrophages.^17^ Elevated sCD163 levels may reflect reduced CD163 expression on these cells.^39^ Several reports have suggested that elevated circulating sCD163 levels are associated with various pathological conditions, including infection,^40^ acute coronary syndromes^41^ and heart failure.^18^ Here we demonstrated that macrophage CD163 is essential for the reparative function of macrophages after MI, promoting cardiac repair and preserving LV function. Furthermore, sCD163 levels are significantly positively associated with the severity of systolic dysfunction and LV dilation in patients with ICM. These findings suggest that increased circulating sCD163 may indicate diminished CD163 expression and impaired macrophage‐mediated cardiac repair, thereby potentially increasing the risk of heart failure following MI. A limitation of the present study is the relatively small sample size. Validation in larger, multicentre cohorts will be necessary to establish the role of sCD163 in heart failure due to ICM.

OPN expression by M2 macrophages has been widely reported.^11^, ^42^, ^43^ Recently, Ding et al. reported that CD163^+^ macrophages contribute to rapid pulmonary fibrosis in COVID‐19 patients by secreting OPN,^44^ Yang et al. demonstrated that CD163^+^ macrophages serve as a significant source of OPN within the tumour microenvironment.^45^ Here, we found that OPN expression is regulated by CD163 in cMacs under both physiological conditions and after MI, as shown by scRNA‐seq and Western blot analyses, which revealed reduced OPN levels in the heart of CD163‐deficient mice in both Sham and MI groups at Day 28 post‐MI. These results suggested that CD163^+^ macrophages are one of the major sources of OPN in various tissues.

For the mechanism, IL‐10 was reported to promote the OPN expression in cultured macrophage via STAT3.^46^ OPN secreted by CD163^+^ macrophages within the tumour microenvironment can trigger its own expression through the STAT3 signalling pathway, establishing a robust autocrine feedback loop.^45^ Here, phospho‐STAT3 levels were not increased in MI‐treated CD163‐deficient mice, suggesting that the STAT3 signalling pathway may be one of mediators through which CD163 regulates OPN expression. Although isolating primary cMacs to investigate the CD163‐mediated regulation of OPN and the JAK‐STAT3 signalling axis is of great importance, such mechanistic studies were constrained by the limited experimental conditions. Meanwhile, we observed that CD163^+^ macrophages are not the only source of OPN in the heart, T cells may also contributes to OPN expression. OPN expression has been reported in both CD4^+^ and CD8^+^ T cells, where it is regulated by the transcription factor T‐bet.^47^ This suggests that an intricate intercellular crosstalk network, involving macrophages, other immune cell subsets and endothelial cells, may exist to coordinately regulate OPN expression.

OPN‐deficient mice exhibit greater LV chamber dilation and impaired systolic function following MI.^35^ Administration of OPN at the border of the ischaemic zone slightly improved systolic function post‐MI, but the effect was not statistically significant, with only a modest reduction in LV diameter.^37^ In contrast, the aggravated systolic dysfunction and ventricular dilation observed in CD163‐deficient mice were significantly alleviated by OPN supplementation. These results suggest that OPN may have a more pronounced therapeutic effect under conditions of CD163 deficiency, which highlights a speculation that OPN therapy might be particularly effective in ICM patients with low macrophage CD163 expression.

TTC staining showed that cardiomyocyte death after MI did not differ between WT and CD163‐deficient mice. However, CD163‐deficient hearts displayed a significantly larger fibrotic scar area, reduced scar thickness and a decreased elastin‐to‐collagen ratio, indicating impaired cardiac repair and excessive scar expansion during the reparative phase. Notably, OPN treatment attenuated scar size, suggesting that OPN promotes cardiac repair in CD163‐deficient hearts. Elastin, an insoluble ECM component composed of a tropoelastin core surrounded by fibrillin and microfibrils, is known to limit infarct expansion and preserve ventricular function when upregulated within the myocardial scar.^8^, ^48^ Consistently, exogenous tropoelastin administration has been shown to improve systolic function.^7^ Moreover, impaired cardiac repair in OPN‐deficient mice has been linked to increased MMP2 and MMP9 activity^49^, ^50^ and reduced collagen deposition in the infarct zone.^35^ In patients with dilated cardiomyopathy, OPN and collagen Types I and III are markedly elevated, with a strong positive association observed between OPN and collagen,^51^ supporting the notion that OPN is essential for sufficient scar formation. However, the relationship between OPN and elastin deposition is not well understood. Thus, the mechanisms underlying the balance of the elastin‐to‐collagen ratio remain largely unclear. Our results suggest that both OPN and CD163 play essential roles in regulating ECM composition in the infarct zone, thereby influencing cardiac repair.

Furthermore, in the remote region, we observed an increase in CD163 expression during the acute phase, consistent with multiple reports showing enhanced macrophage infiltration at this stage, which has been associated with increased chemokine‐mediated recruitment.^4^, ^6^ Although we did not directly assess the functional role of CD163^+^ macrophages in the remote region with respect to cardiac remodelling, previous studies suggest that macrophages in this area contribute to immune modulation, tissue repair, and ECM remodelling outside the infarct zone, at least in part through protease activity.^52^ Our RNA‐seq data further indicate that MI‐induced upregulated genes in Cluster 6 (Figure 6B and Figure S10E) of the remote region are primarily associated with ECM organization. Notably, the expression of these genes was further reduced by both CD163 deficiency and OPN administration, suggesting that macrophage CD163 may also play a role in regulating remote myocardial remodelling. However, whether OPN is directly involved in this process remains unclear.

Interestingly, T‐cell subclustering analysis revealed significant alterations in central memory T cells, including changes in the expression of multiple ECM‐related molecules and OPN. In contrast to HBV‐induced liver failure, where cytotoxic CD8^+^ T cells are functionally impaired and exhaustion is prominent due to sustained antigenic stimulation,^38^ MI represents an acute cell death–driven inflammatory stimulus. In this context, previous studies have shown that depletion of CD8^+^ T cells impairs cardiac repair, at least in part through dysregulated protease activity and defective wound healing.^53^ Therefore, the reduction in Spp1^+^ central memory T cells induced by CD163 deficiency may also contribute to impaired post‐infarction cardiac repair. These findings further suggest the existence of a functional interaction between CD163^+^ macrophages and T cells in regulating myocardial healing. Supporting this concept, recent evidence has demonstrated that TREM2‐expressing macrophages regulate CD8^+^ T‐cell infiltration and function after MI, thereby promoting cardiac repair.^54^ Taken together, our findings raise the possibility that macrophage CD163 may facilitate cardiac repair not only through macrophage‐intrinsic mechanisms but also by modulating adaptive T‐cell responses.

## 5 CONCLUSIONS

Our study demonstrates that macrophage CD163 is critical for cardiac repair after MI, promoting the function of reparative macrophages, reducing LV dilation and improving systolic function through regulation of OPN expression. Circulating sCD163 is elevated in ICM and correlates with the severity of systolic dysfunction and ventricular remodeling. Loss of CD163 impairs macrophage OPN expression, whereas local OPN administration in CD163‐deficient hearts restores systolic performance, reduces scar size and rebalances the elastin‐to‐collagen ratio. These findings highlight the essential role of CD163‐mediated OPN signalling in post‐infarction cardiac repair.

## AUTHOR CONTRIBUTIONS

*Conceptualization*: Jingyu Chen, Linjian Chen, Peng Zhang, Cuilian Dai and Binbin Liu. *Methodology*: Jingyu Chen, Linjian Chen, Wei Huang, Gang Wang, Lin Wang, Wanchun Mei, Wei Ni, Yang Liu and Binbin Liu. *Validation*: Jingyu Chen, Linjian Chen, Wei Huang, Gang Wang, Lin Wang, Wanchun Mei and Wei Ni. *Investigation*: Jingyu Chen, Linjian Chen, Wei Huang, Gang Wang, Lin Wang, Wanchun Mei, Wei Ni, Yang Liu, Licheng Ding, Xiaofeng Ge, Zhaokai Li, Jing Yu, Shufen Huang, Jiayi Lin and Yifan Chen. *Formal analysis*: Wei Huang, Gang Wang and Binbin Liu. *Software*: Gang Wang and Binbin Liu. *Resources*: Licheng Ding, Xiaofeng Ge, Zhaokai Li, Jing Yu, Shufen Huang, Jiayi Lin, Yifan Chen and Binni Cai. *Data curation*: Peng Zhang, Cuilian Dai and Binbin Liu. *Visualization*: Jingyu Chen, Peng Zhang and Binbin Liu. *Writing—original draft*: Jingyu Chen and Binbin Liu. *Writing—review and editing*: Peng Zhang, Cuilian Dai and Binbin Liu. *Supervision*: Binni Cai and Cuilian Dai. *Project administration*: Binbin Liu. *Funding acquisition*: Xiaofeng Ge, Zhaokai Li, Peng Zhang, Cuilian Dai and Binbin Liu.

## CONFLICT OF INTEREST STATEMENT

The authors declare no conflicts of interest.

## FUNDING INFORMATION

National Natural Science Foundation of China (82400406) to P. Zhang; Fujian Natural Science Foundation (2024J011424, 2024J011428, 2025J011494) to B. Liu, Z. Li and P. Zhang; the Xiamen Medical and Health Project (3502Z20224ZD1182, 2024GZL‐GG08) to X. Ge and B. Liu and theNational Key Research and Development Program of China (2019YFE0113900) to C. Dai

## ETHICS STATEMENT

The cross‐sectional study protocol received approval from the Ethics Committee of Xiamen Cardiovascular Hospital, Xiamen University (approval number: 2024YLK33). The mouse study protocol was approved by the Xiamen University Animal Ethics Committee (Protocol No. XMULAC20200150).

## Supporting information



Supporting Information

## Data Availability

The dataset generated in this work was available at GEO: GSE306718. Additional information required to reanalyse the data reported in this paper is available from the lead contact upon request.

## References

[1] Tsao CW , Aday AW , Almarzooq ZI , et al. Heart disease and stroke statistics—2023 update: a report from the American Heart Association. Circulation. 2023;147:e93‐e621. doi:10.1161/CIR.0000000000001123 36695182 10.1161/CIR.0000000000001123 PMC12135016

[2] Cabac‐Pogorevici I , Muk B , Rustamova Y , et al. Ischaemic cardiomyopathy. Pathophysiological insights, diagnostic management and the roles of revascularisation and device treatment. Gaps and dilemmas in the era of advanced technology. Eur J Heart Fail. 2020;22:789‐799. doi:10.1002/ejhf.1747 32020756 10.1002/ejhf.1747

[3] Liga R , Colli A , Taggart DP , et al. Myocardial revascularization in patients with ischemic cardiomyopathy: for whom and how. J Am Heart Assoc. 2023;12:e026943. doi:10.1161/JAHA.122.026943 36892041 10.1161/JAHA.122.026943 PMC10111551

[4] Yap J , Irei J , Lozano‐Gerona J , et al. Macrophages in cardiac remodelling after myocardial infarction. Nat Rev Cardiol. 2023;20:373‐385. doi:10.1038/s41569‐022‐00823‐5 36627513 10.1038/s41569-022-00823-5

[5] Shinde AV , Frangogiannis NG . Fibroblasts in myocardial infarction: a role in inflammation and repair. J Mol Cell Cardiol. 2014;70:74‐82. doi:10.1016/j.yjmcc.2013.11.015 24321195 10.1016/j.yjmcc.2013.11.015 PMC3995820

[6] Prabhu SD , Frangogiannis NG . The biological basis for cardiac repair after myocardial infarction: from inflammation to fibrosis. Circ Res. 2016;119:91‐112. doi:10.1161/CIRCRESAHA.116.303577 27340270 10.1161/CIRCRESAHA.116.303577 PMC4922528

[7] Hume RD , Kanagalingam S , Deshmukh T , et al. Tropoelastin improves post‐infarct cardiac function. Circ Res. 2023;132:72‐86. doi:10.1161/CIRCRESAHA.122.321123 36453283 10.1161/CIRCRESAHA.122.321123 PMC9829044

[8] Mizuno T , Yau TM , Weisel RD , et al. Elastin stabilizes an infarct and preserves ventricular function. Circulation. 2005;112:I81‐88. doi:10.1161/01.CIRCULATIONAHA.105.523795 16159870 10.1161/01.CIRCULATIONAHA.105.523795

[9] Bajpai G , Bredemeyer A , Li W , et al. Tissue resident CCR2− and CCR2+ cardiac macrophages differentially orchestrate monocyte recruitment and fate specification following myocardial injury. Circ Res. 2019;124:263‐278. doi:10.1161/CIRCRESAHA.118.314028 30582448 10.1161/CIRCRESAHA.118.314028 PMC6626616

[10] Leblond A‐L , Klinkert K , Martin K , et al. Systemic and cardiac depletion of M2 macrophage through CSF‐1R signaling inhibition alters cardiac function post myocardial infarction. PLoS One. 2015;10:e0137515. doi:10.1371/journal.pone.0137515 26407006 10.1371/journal.pone.0137515 PMC4583226

[11] Shiraishi M , Shintani Y , Shintani Y , et al. Alternatively activated macrophages determine repair of the infarcted adult murine heart. J Clin Invest. 2016;126:2151‐2166. doi:10.1172/JCI85782 27140396 10.1172/JCI85782 PMC4887176

[12] Chen R , Zhang H , Tang B , et al. Macrophages in cardiovascular diseases: molecular mechanisms and therapeutic targets. Signal Transduct Target Ther. 2024;9:130. doi:10.1038/s41392‐024‐01840‐1 38816371 10.1038/s41392-024-01840-1 PMC11139930

[13] Humeres C , Frangogiannis NG . Fibroblasts in the infarcted, remodeling, and failing heart. JACC Basic Transl Sci. 2019;4:449‐467. doi:10.1016/j.jacbts.2019.02.006 31312768 10.1016/j.jacbts.2019.02.006 PMC6610002

[14] Zhang S , Zhang Y , Duan X , et al. Targeting NPM1 epigenetically promotes postinfarction cardiac repair by reprogramming reparative macrophage metabolism. Circulation. 2024;149:1982‐2001. doi:10.1161/CIRCULATIONAHA.123.065506 38390737 10.1161/CIRCULATIONAHA.123.065506 PMC11175795

[15] Wang Q , Ismahil MA , Zhu Y , et al. CD206+IL‐4Rα+ macrophages are drivers of adverse cardiac remodeling in ischemic cardiomyopathy. Circulation. 2025;152:257‐273. doi:10.1161/CIRCULATIONAHA.124.072411 40308203 10.1161/CIRCULATIONAHA.124.072411 PMC12303760

[16] Chen L , Mei W , Song J , et al. CD163 protein inhibits lipopolysaccharide‐induced macrophage transformation from M2 to M1 involved in disruption of the TWEAK‐Fn14 interaction. Heliyon. 2024;10:e23223. doi:10.1016/j.heliyon.2023.e23223 38148798 10.1016/j.heliyon.2023.e23223 PMC10750081

[17] Etzerodt A , Rasmussen MR , Svendsen P , et al. Structural basis for inflammation‐driven shedding of CD163 ectodomain and tumor necrosis factor‐α in macrophages. J Biol Chem. 2014;289:778‐788. doi:10.1074/jbc.M113.520213 24275664 10.1074/jbc.M113.520213 PMC3887204

[18] Durda P , Raffield LM , Lange EM , et al. Circulating soluble CD163, associations with cardiovascular outcomes and mortality, and identification of genetic variants in older individuals: the Cardiovascular Health Study. J Am Heart Assoc. 2022;11:e024374. doi:10.1161/JAHA.121.024374 36314488 10.1161/JAHA.121.024374 PMC9673628

[19] Ni W , Ge X , Liu Y , et al. CD163+ macrophages attenuate pressure overload‐induced left ventricular systolic dysfunction and cardiac mitochondrial dysfunction via interleukin‐10. Basic Res Cardiol. 2025;120:727‐744. doi:10.1007/s00395‐025‐01114‐z 40343453 10.1007/s00395-025-01114-z

[20] Dai C , Yao X , Gordon EM , et al. A CCL24‐dependent pathway augments eosinophilic airway inflammation in house dust mite‐challenged Cd163−/− mice. Mucosal Immunol. 2016;9:702‐717. doi:10.1038/mi.2015.94 26376364 10.1038/mi.2015.94 PMC4794428

[21] Gao E , Lei YH , Shang X , et al. A novel and efficient model of coronary artery ligation and myocardial infarction in the mouse. Circ Res. 2010;107:1445‐1453. doi:10.1161/CIRCRESAHA.110.223925 20966393 10.1161/CIRCRESAHA.110.223925 PMC3005817

[22] Kucherenko MM , Sang P , Yao J , et al. Elastin stabilization prevents impaired biomechanics in human pulmonary arteries and pulmonary hypertension in rats with left heart disease. Nat Commun. 2023;14:4416. doi:10.1038/s41467‐023‐39934‐z 37479718 10.1038/s41467-023-39934-z PMC10362055

[23] Schindelin J , Arganda‐Carreras I , Frise E , et al. Fiji: an open‐source platform for biological‐image analysis. Nat Methods. 2012;9:676‐682. doi:10.1038/nmeth.2019 22743772 10.1038/nmeth.2019 PMC3855844

[24] Liu B , Maekawa T , Yoshida K , et al. Telomere shortening by transgenerational transmission of TNF‐α‐induced TERRA via ATF7. Nucleic Acids Res. 2019;47:283‐298. doi:10.1093/nar/gky1149 30407559 10.1093/nar/gky1149 PMC6326783

[25] Kim D , Paggi JM , Park C , et al. Graph‐based genome alignment and genotyping with HISAT2 and HISAT‐genotype. Nat Biotechnol. 2019;37:907‐915. doi:10.1038/s41587‐019‐0201‐4 31375807 10.1038/s41587-019-0201-4 PMC7605509

[26] Liao Y , Smyth GK , Shi W . featureCounts: an efficient general purpose program for assigning sequence reads to genomic features. Bioinformatics. 2014;30:923‐930. doi:10.1093/bioinformatics/btt656 24227677 10.1093/bioinformatics/btt656

[27] McEvoy JW , McCarthy CP , Bruno RM , et al. 2024 ESC Guidelines for the management of elevated blood pressure and hypertension. Eur Heart J. 2024;45:3912‐4018. doi:10.1093/eurheartj/ehae178 39210715 10.1093/eurheartj/ehae178

[28] Rickham PP . Human experimentation. Code of ethics of the world medical association. Declaration of Helsinki. Br Med J. 1964;2:177. doi:10.1136/bmj.2.5402.177 14150898 10.1136/bmj.2.5402.177 PMC1816102

[29] Gaasch WH , Zile MR . Left ventricular structural remodeling in health and disease: with special emphasis on volume, mass, and geometry. J Am Coll Cardiol. 2011;58:1733‐1740. doi:10.1016/j.jacc.2011.07.022 21996383 10.1016/j.jacc.2011.07.022

[30] Etzerodt A , Moestrup SK . CD163 and inflammation: biological, diagnostic, and therapeutic aspects. Antioxid Redox Signal. 2013;18:2352‐2363. doi:10.1089/ars.2012.4834 22900885 10.1089/ars.2012.4834 PMC3638564

[31] Smiseth OA , Torp H , Opdahl A , et al. Myocardial strain imaging: how useful is it in clinical decision making? Eur Heart J. 2016;37:1196‐1207. doi:10.1093/eurheartj/ehv529 26508168 10.1093/eurheartj/ehv529 PMC4830908

[32] Zhou RX , Higgins MK . Scavenger receptor CD163 multimerises to allow uptake of diverse ligands. Nat Commun. 2025;16:6623. doi:10.1038/s41467‐025‐62054‐9 40681524 10.1038/s41467-025-62054-9 PMC12274614

[33] De Bei O , Campanini B . Strength in numbers: how multimerization drives HpHb uptake by CD163 scavenging receptor. Nat Commun. 2025;17:111. doi:10.1038/s41467‐025‐67812‐3 41444480 10.1038/s41467-025-67812-3 PMC12774901

[34] Dick SA , Macklin JA , Nejat S , et al. Self‐renewing resident cardiac macrophages limit adverse remodeling following myocardial infarction. Nat Immunol. 2019;20:29‐39. doi:10.1038/s41590‐018‐0272‐2 30538339 10.1038/s41590-018-0272-2 PMC6565365

[35] Trueblood NA , Xie Z , Communal C , et al. Exaggerated left ventricular dilation and reduced collagen deposition after myocardial infarction in mice lacking osteopontin. Circ Res. 2001;88:1080‐1087. doi:10.1161/hh1001.090842 11375279 10.1161/hh1001.090842

[36] Santos‐Zas I , Lemarié J , Zlatanova I , et al. Cytotoxic CD8+ T cells promote granzyme B‐dependent adverse post‐ischemic cardiac remodeling. Nat Commun. 2021;12:1483. doi:10.1038/s41467‐021‐21737‐9 33674611 10.1038/s41467-021-21737-9 PMC7935973

[37] Rotem I , Konfino T , Caller T , et al. Osteopontin promotes infarct repair. Basic Res Cardiol. 2022;117:51. doi:10.1007/s00395‐022‐00957‐0 36239866 10.1007/s00395-022-00957-0

[38] Liang X , Luo J , Zhou Q , et al. Single‐cell multimodal analysis reveals the dynamic immunopathogenesis of HBV‐ACLF progression. Gut. 2026;75:367‐381. doi:10.1136/gutjnl‐2024‐333308 40841166 10.1136/gutjnl-2024-333308 PMC12916473

[39] Hergel LLF , Kielsen K , Weischendorff S , et al. The macrophage activation marker sCD163 in acute and chronic graft‐versus‐host disease after pediatric hematopoietic stem cell transplantation. Bone Marrow Transplant. 2023;58:587‐589. doi:10.1038/s41409‐023‐01927‐3 36721043 10.1038/s41409-023-01927-3

[40] Yap Y‐J , Wong P‐F , AbuBakar S , et al. The clinical utility of CD163 in viral diseases. Clin Chim Acta. 2023;541:117243. doi:10.1016/j.cca.2023.117243 36740088 10.1016/j.cca.2023.117243

[41] Plevriti A , Lamprou M , Mourkogianni E , et al. The role of soluble CD163 (sCD163) in human physiology and pathophysiology. Cells. 2024;13:1679. doi:10.3390/cells13201679 39451197 10.3390/cells13201679 PMC11506427

[42] Schack L , Stapulionis R , Christensen B , et al. Osteopontin enhances phagocytosis through a novel osteopontin receptor, the alphaXbeta2 integrin. J Immunol. 2009;182:6943‐6950. doi:10.4049/jimmunol.0900065 19454691 10.4049/jimmunol.0900065

[43] Shin Y‐J , Kim HL , Choi J‐S , et al. Osteopontin: correlation with phagocytosis by brain macrophages in a rat model of stroke. Glia. 2011;59:413‐423. doi:10.1002/glia.21110 21264948 10.1002/glia.21110

[44] Ding W , Deng S , Wang Z , et al. CD163+ macrophages drive rapid pulmonary fibrosis via osteopontin secretion. Int Immunopharmacol. 2025;161:114976. doi:10.1016/j.intimp.2025.114976 40466617 10.1016/j.intimp.2025.114976

[45] Yang Y‐E , Lin Y‐A , Ling L‐L , et al. Rab37‐mediated OPN secretion enriches SPP1+ macrophages through autocrine‐paracrine signaling to drive lung tumor progression. Oncogenesis. 2026;15:4. doi:10.1038/s41389‐026‐00596‐3 41535255 10.1038/s41389-026-00596-3 PMC12823564

[46] Shirakawa K , Endo J , Kataoka M , et al. IL (interleukin)‐10‐STAT3‐galectin‐3 axis is essential for osteopontin‐producing reparative macrophage polarization after myocardial infarction. Circulation. 2018;138:2021‐2035. doi:10.1161/CIRCULATIONAHA.118.035047 29967195 10.1161/CIRCULATIONAHA.118.035047

[47] Shinohara ML , Jansson M , Hwang ES , et al. T‐bet‐dependent expression of osteopontin contributes to T cell polarization. Proc Natl Acad Sci USA. 2005;102:17101‐17106. doi:10.1073/pnas.0508666102 16286640 10.1073/pnas.0508666102 PMC1288014

[48] Lichtenauer M , Mildner M , Baumgartner A , et al. Intravenous and intramyocardial injection of apoptotic white blood cell suspensions prevents ventricular remodelling by increasing elastin expression in cardiac scar tissue after myocardial infarction. Basic Res Cardiol. 2011;106:645‐655. doi:10.1007/s00395‐011‐0173‐0 21416207 10.1007/s00395-011-0173-0 PMC3105227

[49] Xie Z , Singh M , Siwik DA , et al. Osteopontin inhibits interleukin‐1beta‐stimulated increases in matrix metalloproteinase activity in adult rat cardiac fibroblasts: role of protein kinase C‐zeta. J Biol Chem. 2003;278:48546‐48552. doi:10.1074/jbc.M302727200 14500723 10.1074/jbc.M302727200

[50] Krishnamurthy P , Peterson JT , Subramanian V , et al. Inhibition of matrix metalloproteinases improves left ventricular function in mice lacking osteopontin after myocardial infarction. Mol Cell Biochem. 2009;322:53‐62. doi:10.1007/s11010‐008‐9939‐6 18979185 10.1007/s11010-008-9939-6 PMC2711544

[51] Satoh M , Nakamura M , Akatsu T , et al. Myocardial osteopontin expression is associated with collagen fibrillogenesis in human dilated cardiomyopathy. Eur J Heart Fail. 2005;7:755‐762. doi:10.1016/j.ejheart.2004.10.019 16087132 10.1016/j.ejheart.2004.10.019

[52] Frantz S , Nahrendorf M . Cardiac macrophages and their role in ischaemic heart disease. Cardiovasc Res. 2014;102:240‐248. doi:10.1093/cvr/cvu025 24501331 10.1093/cvr/cvu025 PMC3989449

[53] Ilatovskaya DV , Pitts C , Clayton J , et al. CD8+ T‐cells negatively regulate inflammation post‐myocardial infarction. Am J Physiol Heart Circ Physiol. 2019;317:H581‐H596. doi:10.1152/ajpheart.00112.2019 31322426 10.1152/ajpheart.00112.2019 PMC6766723

[54] Zhang L , Wang S , Ding Y , et al. Macrophage‐TREM2 promotes cardiac repair by restricting the infiltration of CD8+ T cells via CXCL16‐CXCR6 axis after myocardial infarction. Theranostics. 2025;15:9580‐9600. doi:10.7150/thno.118014 41041054 10.7150/thno.118014 PMC12486271

