# Supplementary material for: Macrophage Cluster of Differentiation 163 promotes post‐infarction cardiac repair and preserves left ventricular function via osteopontin
Source: Clin Transl Med. 2026 Apr 23;16(4):e70662. doi: 10.1002/ctm2.70662 (PMC13106882; doi:10.1002/ctm2.70662)
Supplement: Supplementary file 1 — Supporting Information [file CTM2-16-e70662-s001.docx]

Supplementary information

**Macrophage CD163 Promotes Post-Infarction Cardiac Repair and Preserves Left Ventricular Function via Osteopontin**

Jingyu Chen^1, #^, Linjian Chen^1, #^, Wei Huang^1, #^, Gang Wang^1,2, #^, Lin Wang^1^, Wanchun Mei^1^, Wei Ni^3^, Yang Liu^1^, Licheng Ding^1^, Xiaofeng Ge^1^, Zhaokai Li^1^, Jing Yu^1^, Shufen Huang^1^, Jiayi Lin^1^, Yifan Chen^1^, Binni Cai^1^, Peng Zhang^1*^, Cuilian Dai^1*^, Binbin Liu^1*^

1. Xiamen Cardiovascular Hospital, School of Medicine, Xiamen University, Jinshan Road 2999, Xiamen, 361015, China.
2. Department of Cardiology, The Second Affiliated Hospital, Chongqing Medical University, Chongqing, China.
3. Sir Run Run Shaw Hospital, School of Medicine, Zhejiang University, Hangzhou, Qingchun East Road 3, 310016, China.

*Corresponding author at: Xiamen Cardiovascular Hospital, School of Medicine, Xiamen University, Jinshan Road 2999, Xiamen, 361015, China. Phone: +86-0592-2292705; E-mail: [liubinbin@xmu.edu.cn](mailto:liubinbin@xmu.edu.cn) (B. Liu, Leading contact); daicl@xmu.edu.cn (C. Dai); [rocroc313@sina.com](mailto:rocroc313@sina.com) (P. Zhang)

# These authors contributed equally

Supplementary Figures

**
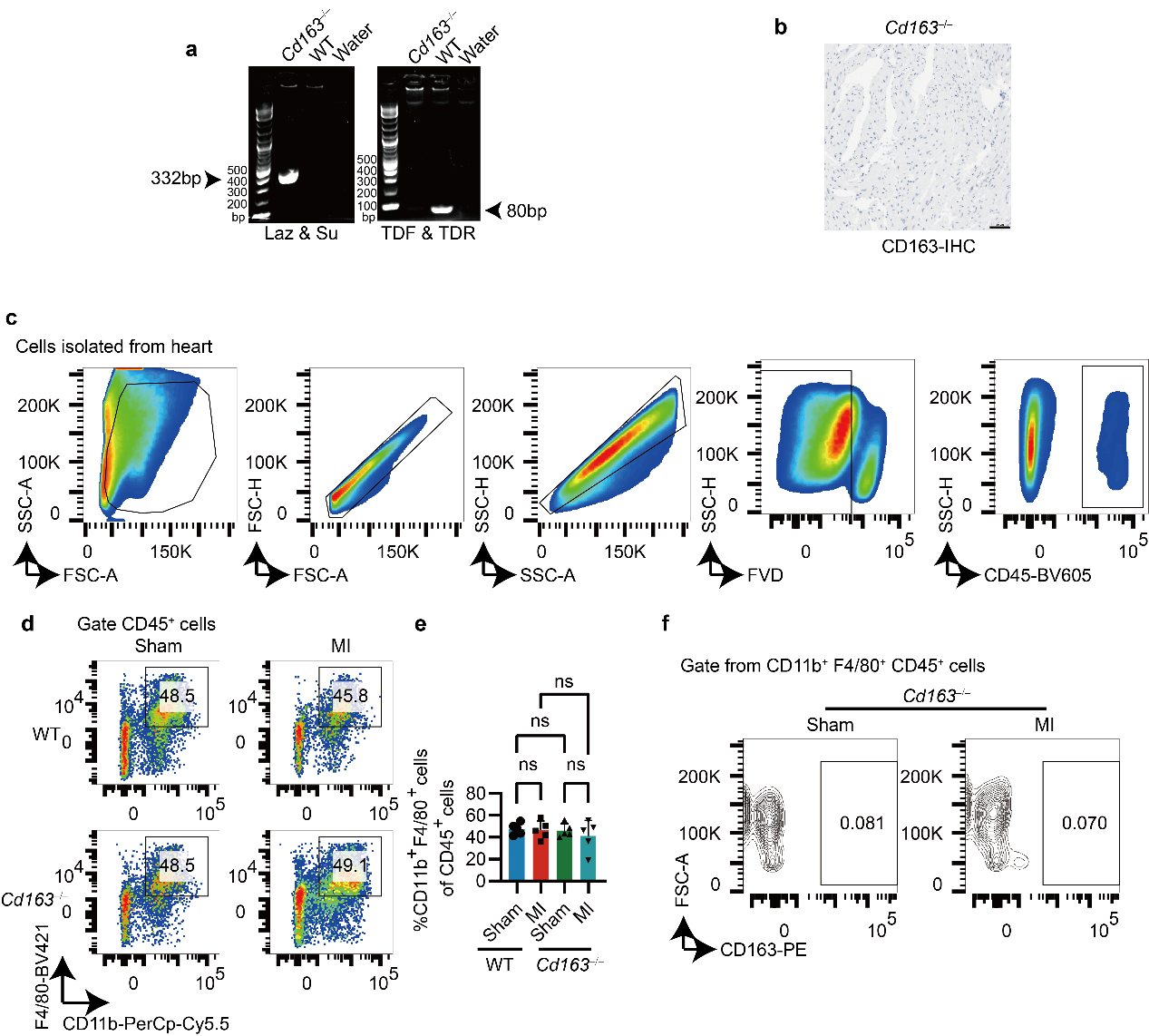
**

**Figure S1. Percentages of cardiac macrophages after MI.** **(a)** Representative PCR results for genotyping. **(b)** Immunohistochemistry of CD163 in the hearts of *Cd163*^−^*^/^*^−^ mice as a negative control. **(c)** Representative flow cytometry gating strategy for CD45⁺ cells from the heart. **(d)** Representative flow cytometry plot showing cardiac macrophages (cMacs). **(e)** Percentage of cMacs among CD45⁺ cells. Data represent individual mice (n = 5) from two experiments, statistics by one-way ANOVA with Tukey’s test. **(f)** Representative flow cytometry plot showing CD163 expression in cMacs from the hearts of *Cd163*^−^*^/^*^−^ mice as a negative control.

**
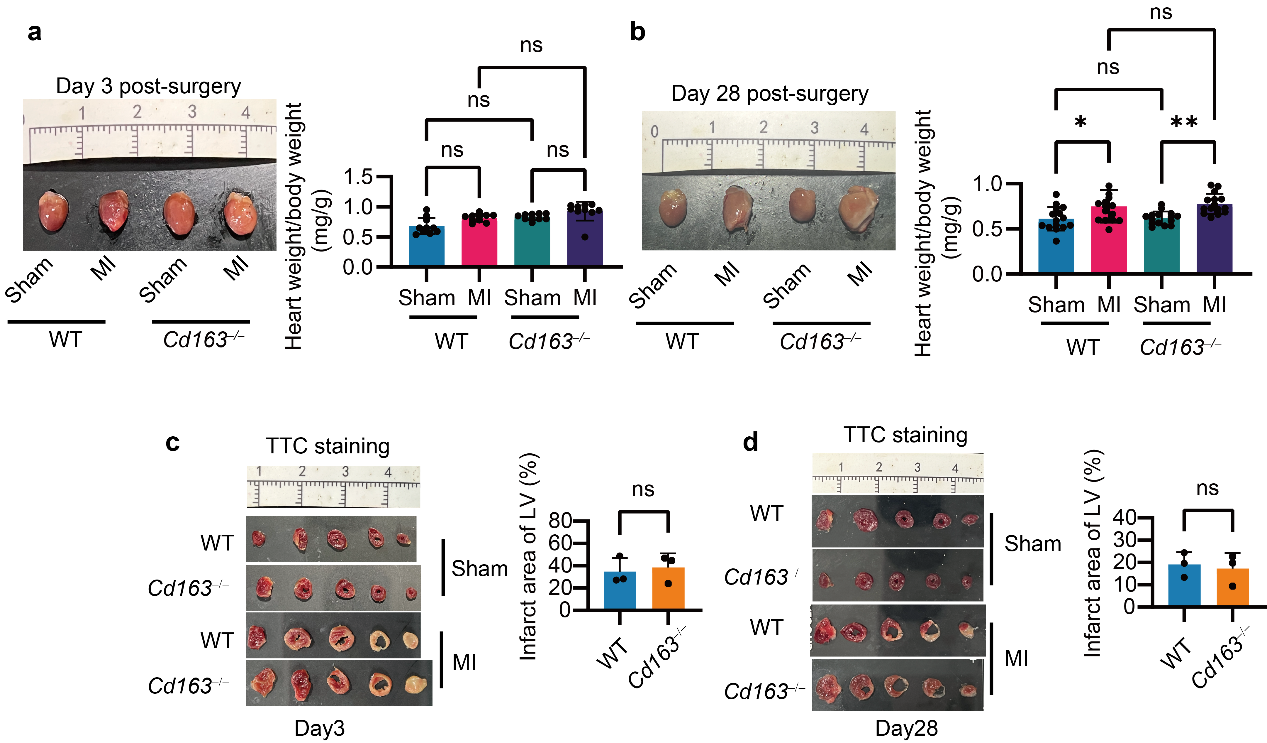
**

**Figure S2. Anatomical findings in WT and *Cd163*^−^*^/^*^−^ mice post-MI.** **(a)** Representative anatomical images of hearts at day 3 and quantification of heart weight to body weight ratio. Data points represent individual mice (n = 10) from two experiments; statistical analysis was performed by Kruskal–Wallis test with Dunn’s multiple comparisons test. **(b)** Representative anatomical images of hearts at day 28 and quantification of heart weight to body weight ratio. Data points represent individual mice (n = 15) from three experiments; statistical analysis was performed by one-way ANOVA with Tukey’s multiple comparisons test. TTC-stained heart sections at **(c)** day 3 and **(d)** day 28 post-MI. Representative images and the quantification of infarct area as a percentage of the LV are shown**.** Data point represents an individual mouse (n = 3) from 2 independent experiments. Statistical analysis was performed using an unpaired t-test.


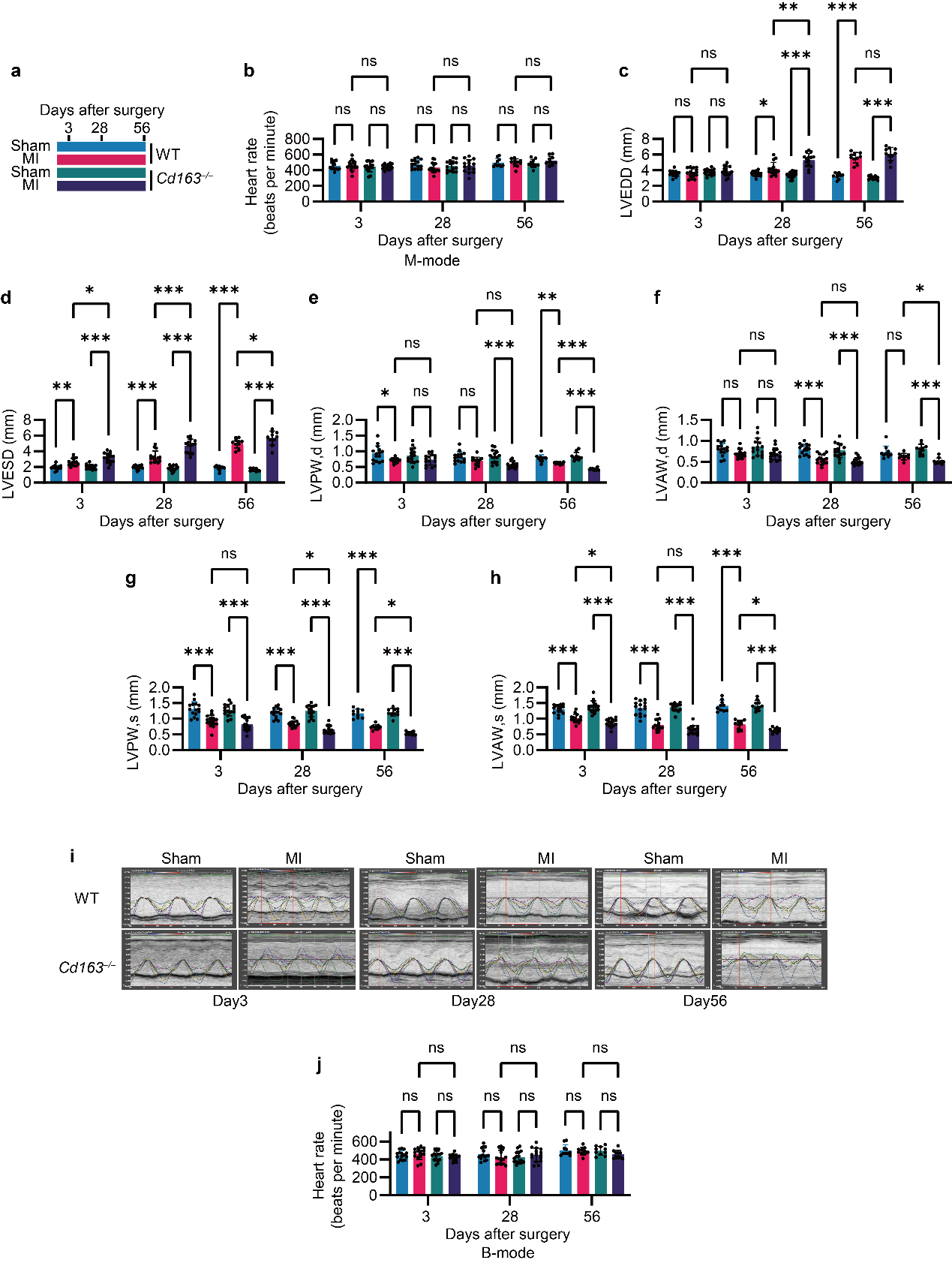


**Figure S3. Echocardiographic findings in WT and *Cd163*^−^*^/^*^−^ mice post-MI. (a)** Schematic diagram of the mouse study design by using WT and *Cd163*^−^*^/^*^−^ mice subjected to LAD ligation or sham surgery. Echocardiographic measurements were obtained from the parasternal long-axis (PLAX) view. **(b)** Heart rate during M-mode imaging; **(c)** left ventricular end-diastolic diameter (LVEDD); **(d)** left ventricular end-systolic diameter (LVESD); **(e)** left ventricular posterior wall thickness at end-diastole (LVPW,d); **(f)** left ventricular anterior wall thickness at end-diastole (LVAW,d); **(g)** left ventricular posterior wall thickness at end-systole (LVPW,s); and **(h)** left ventricular anterior wall thickness at end-systole (LVAW,s). **(i)** Representative echocardiographic images of global longitudinal strain. **(j)** Heart rate during B-mode imaging. Each dot represents one mouse (n = 15 for day 3 and day 28; n = 10 for day 56) pooled from 2-3 independent experiments. Statistical analysis was performed using two-way ANOVA followed by Tukey’s multiple comparisons test.


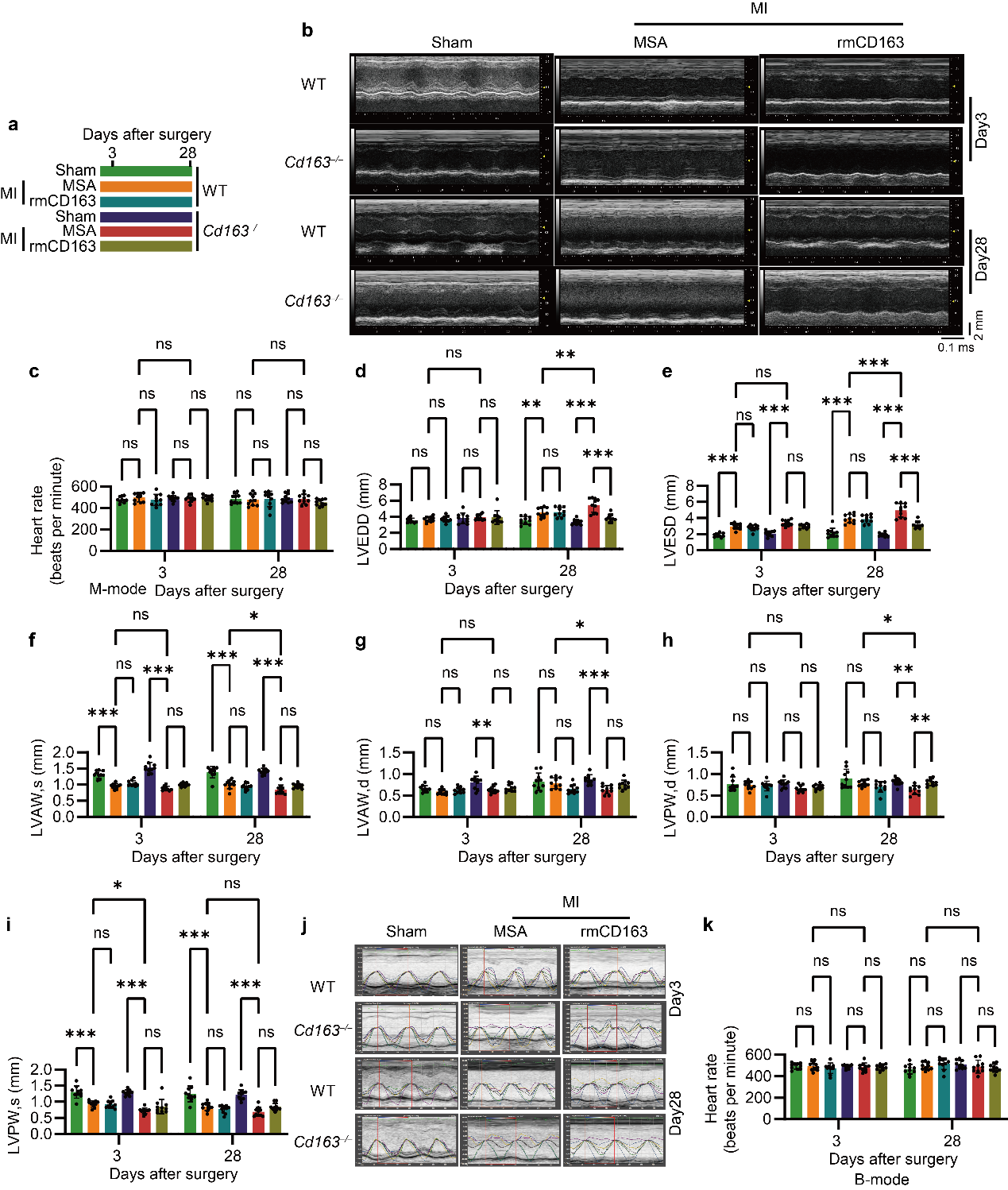


**Figure S4. Echocardiographic findings in WT and *Cd163*^−^*^/^*^−^ mice with or without recombinant mouse CD163 protein treatment post-MI. (a)** Schematic diagram of the mouse study design. Echocardiographic measurements were obtained from the parasternal long-axis (PLAX) view. **(b)** Representative M-mode echocardiography images analyzed by PLAX. **(c)** Heart rate during M-mode imaging; **(d)** left ventricular end-diastolic diameter (LVEDD); **(e)** left ventricular end-systolic diameter (LVESD); **(f)** left ventricular posterior wall thickness at end-systole (LVPW,s); **(g)** left ventricular anterior wall thickness at end-diastole (LVAW,d); **(h)** left ventricular posterior wall thickness at end-diastole (LVPW,d); and **(i)** left ventricular anterior wall thickness at end-systole (LVAW,s). **(j)** Representative echocardiographic images of global longitudinal strain. **(k)** Heart rate during B-mode imaging. Each dot represents one mouse (n = 10) pooled from 2 independent experiments. Statistical analysis was performed using two-way ANOVA followed by Tukey’s multiple comparisons test.


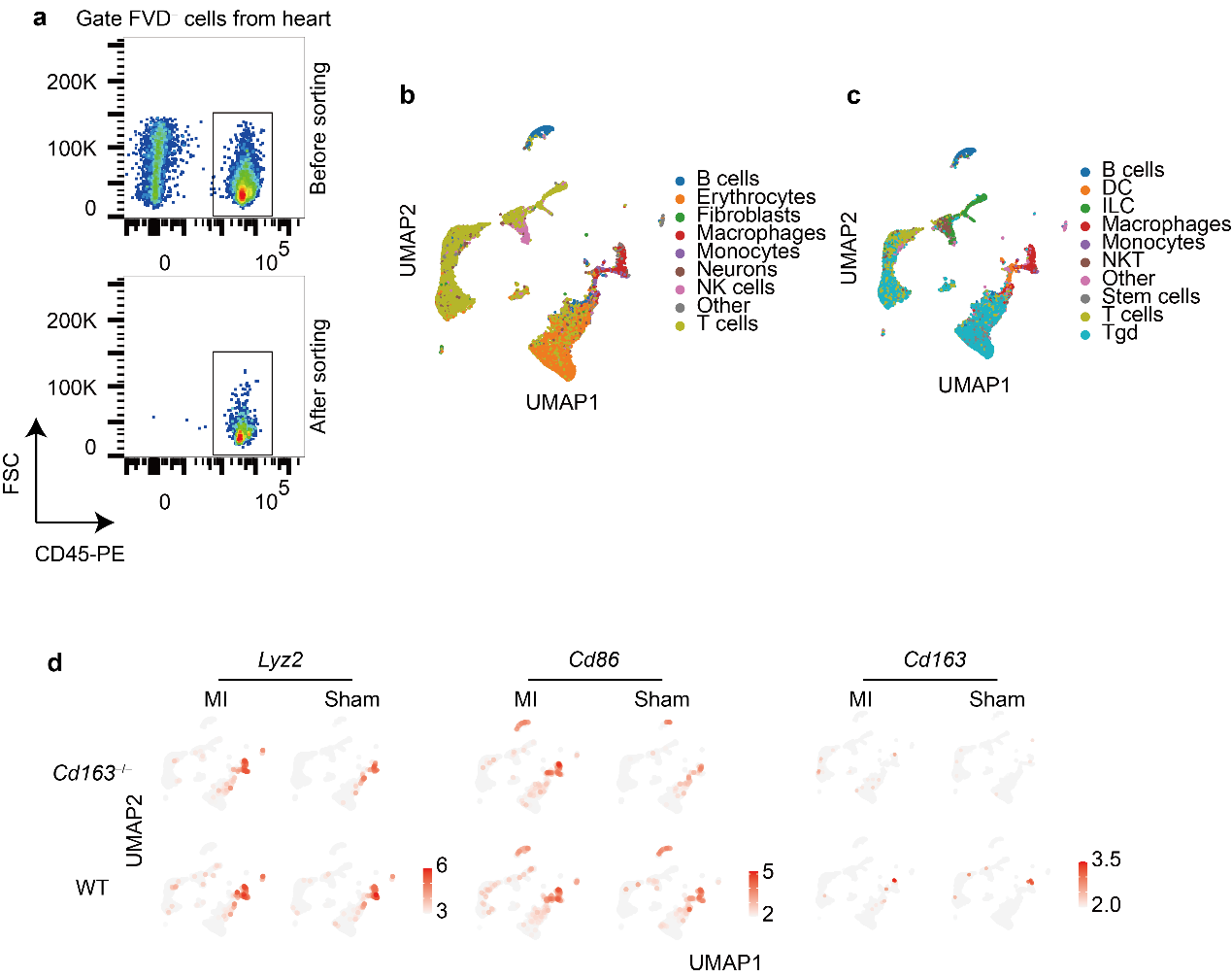


**Figure S5. Clustering of cardiac CD45⁺ cells by scRNA-seq. (a)** CD45⁺ cells were sorted from the heart. Representative flow cytometry plots showing CD45⁺ cells before and after sorting. **(b–c)** A total of 9,335, 9,450, 7,144, and 8,776 CD45⁺ cells were filtered, retained for clustering analysis, and annotated from sham or MI-treated WT and *Cd163*^−^*^/^*^−^ mice. **(b)** Clusters annotated using celldex::MouseRNAseqData. **(c)** Clusters annotated using celldex::ImmGenData. **(d)** Distribution of macrophage marker genes, including *Lyz2*, *Cd86*, and *Cd163*, in CD45⁺ cells.

**
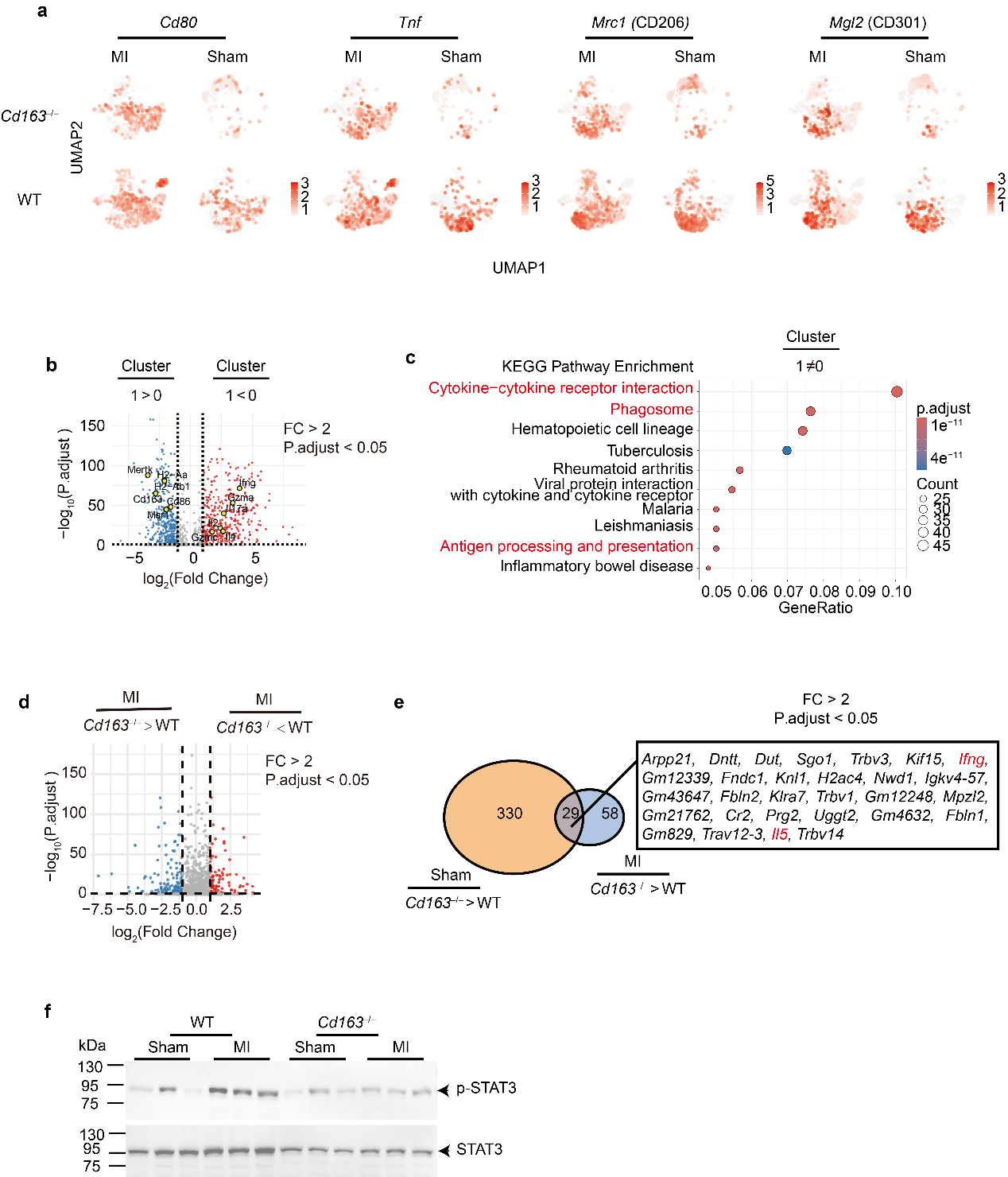
**

**Figure S6. CD163 deficiency did not alter the expression of marker genes related to M1 and M2 macrophages analyzed by scRNA-seq. (a)** Distribution of M1- and M2-associated marker genes in macrophages. **(b)** Volcano plot showing differentially expressed genes between Cluster 0 and 1 in macrophages (cut-off: FC > 2 and adjusted P < 0.05). **(c)** KEGG pathway enrichment analysis of differentially expressed genes (DEGs) between clusters 0 and 1. **(d)** Volcano plot showing differentially expressed genes between WT and *Cd163*^−^*^/^*^−^ mice after MI (cut-off: FC > 2 and adjusted P < 0.05). **(e)** Venn diagram showing CD163 deficiency–induced upregulated genes compared to WT in both sham and MI conditions. **(f)** Protein levels of STAT3 and p-STAT3 Y705 at day 28 post-surgery with internal control as shown in Figure 4l.

**
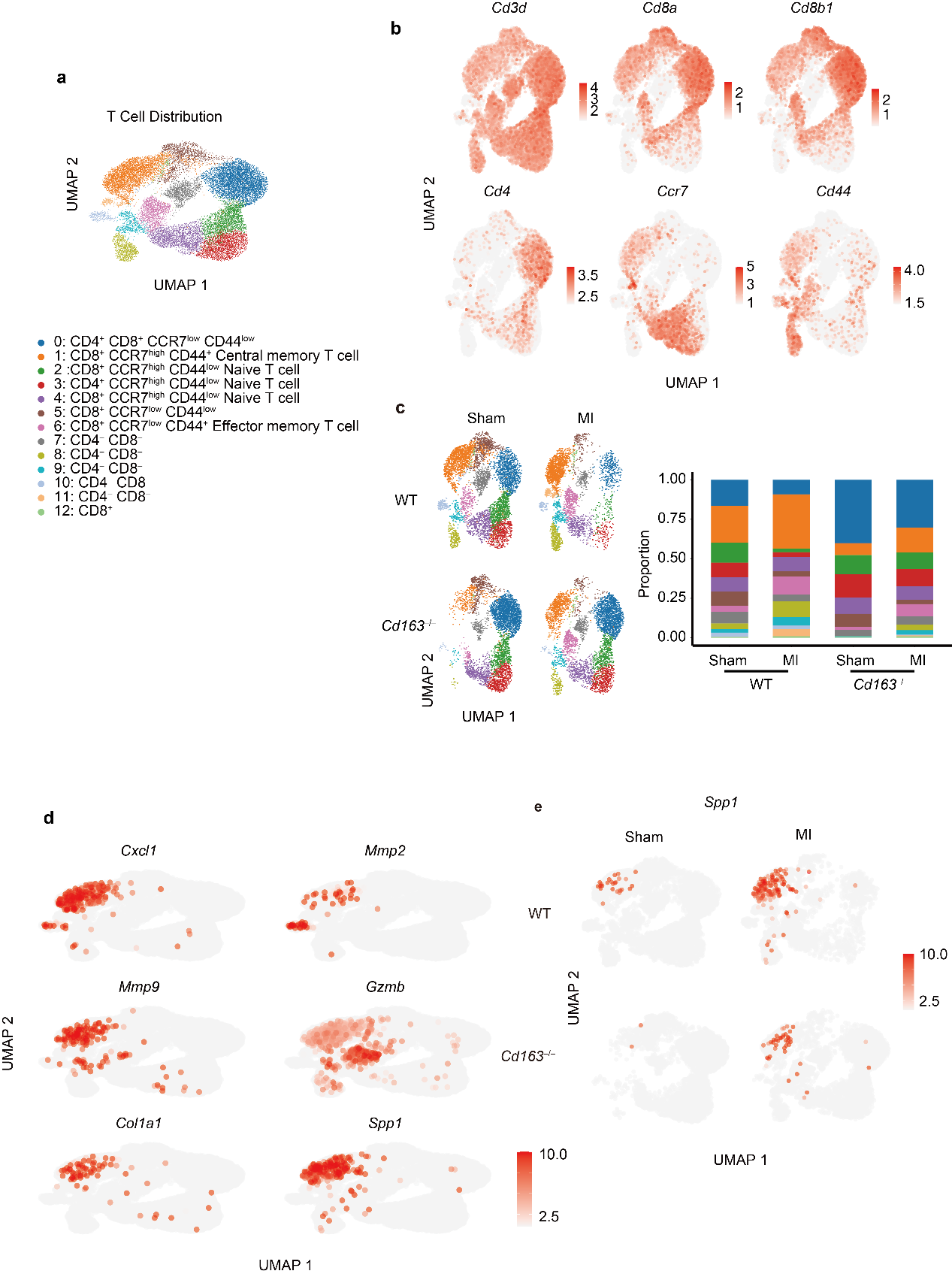
**

**Figure S7. Subclustering analysis of T cells.** T cells were extracted based on the cell annotations generated using celldex::MouseRNAseqData. **(a)** UMAP showing the distribution of T cell subclusters. **(b)** Expression patterns of representative marker genes across T cell subclusters. **(c)** UMAP showing the distribution of T cell subclusters in sham- and MI-treated WT and *Cd163*^−^*^/^*^−^ mice. The proportions of the indicated clusters in each group are also shown. **(d)** Expression distribution of representative genes in T cells. **(e)** Distribution of Spp1 expression in sham- and MI-treated WT and *Cd163*^−^*^/^*^−^ mice.


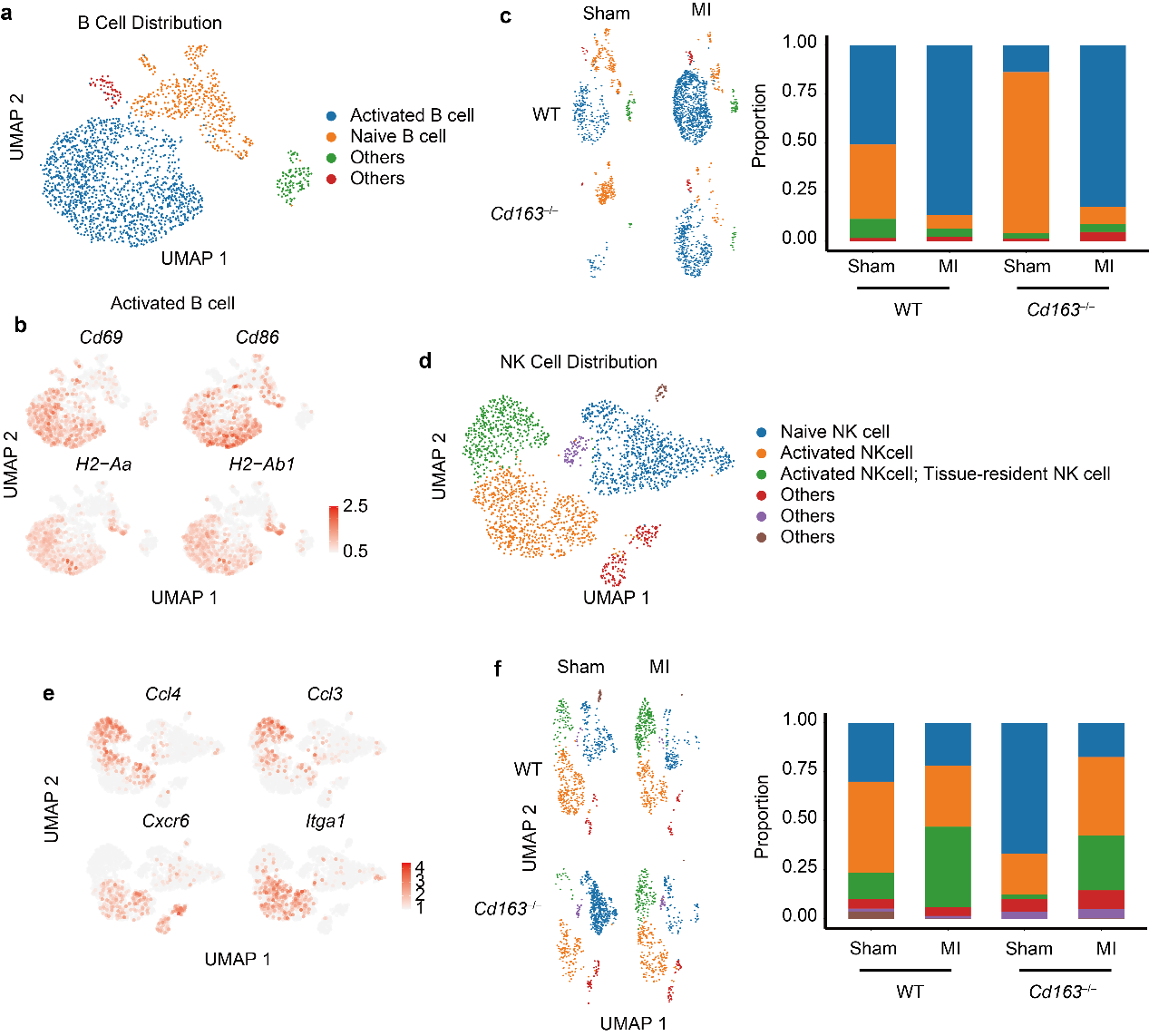


**Figure S8. Subclustering analysis of B cells and NK cells.** The indicating cells were extracted based on the cell annotations generated using celldex::MouseRNAseqData. UMAP showing the distribution of **(a)** B cell and **(d)** NK cell subclusters. Expression patterns of representative marker genes across **(b)** B cell and **(e)** NK cell subclusters. UMAP showing the distribution of **(c)** B cell and **(f)** NK cell subclusters in sham- and MI-treated WT and *Cd163*^−^*^/^*^−^ mice. The proportions of the indicated clusters in each group are also shown.


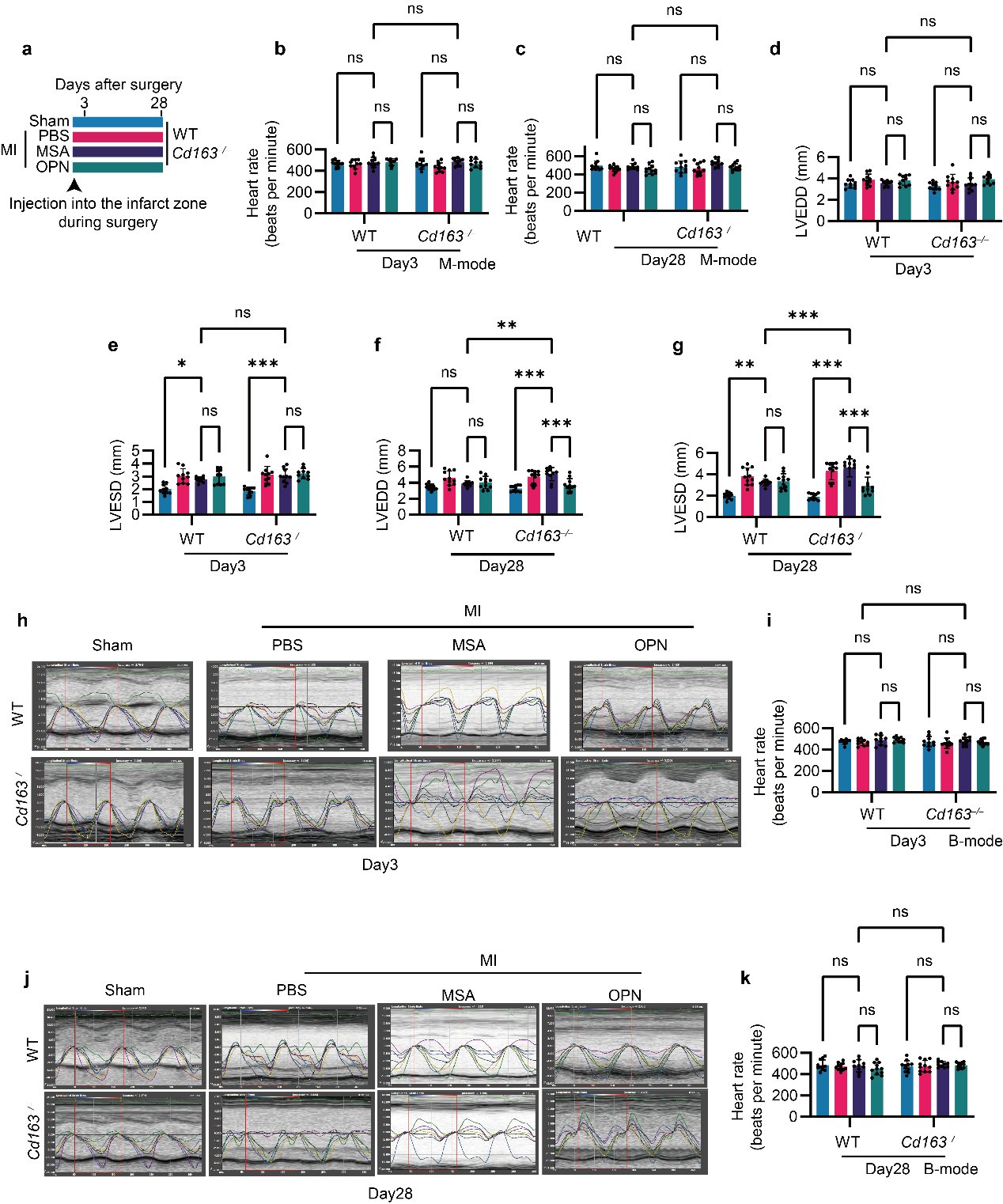


**Figure S9. Echocardiographic findings in WT and *Cd163*^−^*^/^*^−^ mice with or without OPN administration post-MI. (a)** Schematic diagram of the mouse study design. Echocardiographic measurements were obtained from the parasternal long-axis (PLAX) view. **(b–g)** Heart rate during M-mode, left ventricular end-diastolic diameter (LVEDD), and left ventricular end-systolic diameter (LVESD), at day 3 or day 28 post-MI. Representative global longitudinal strain (GLS) images at **(h)** day 3 or **(j)** day 28post-MI. Heart rate during B-mode measurements at **(i** day 3 and **(k)** day 28. Data points represent individual mice (n = 10) from two experiments; statistical analysis was performed by two-way ANOVA with Tukey’s multiple comparisons test.

**
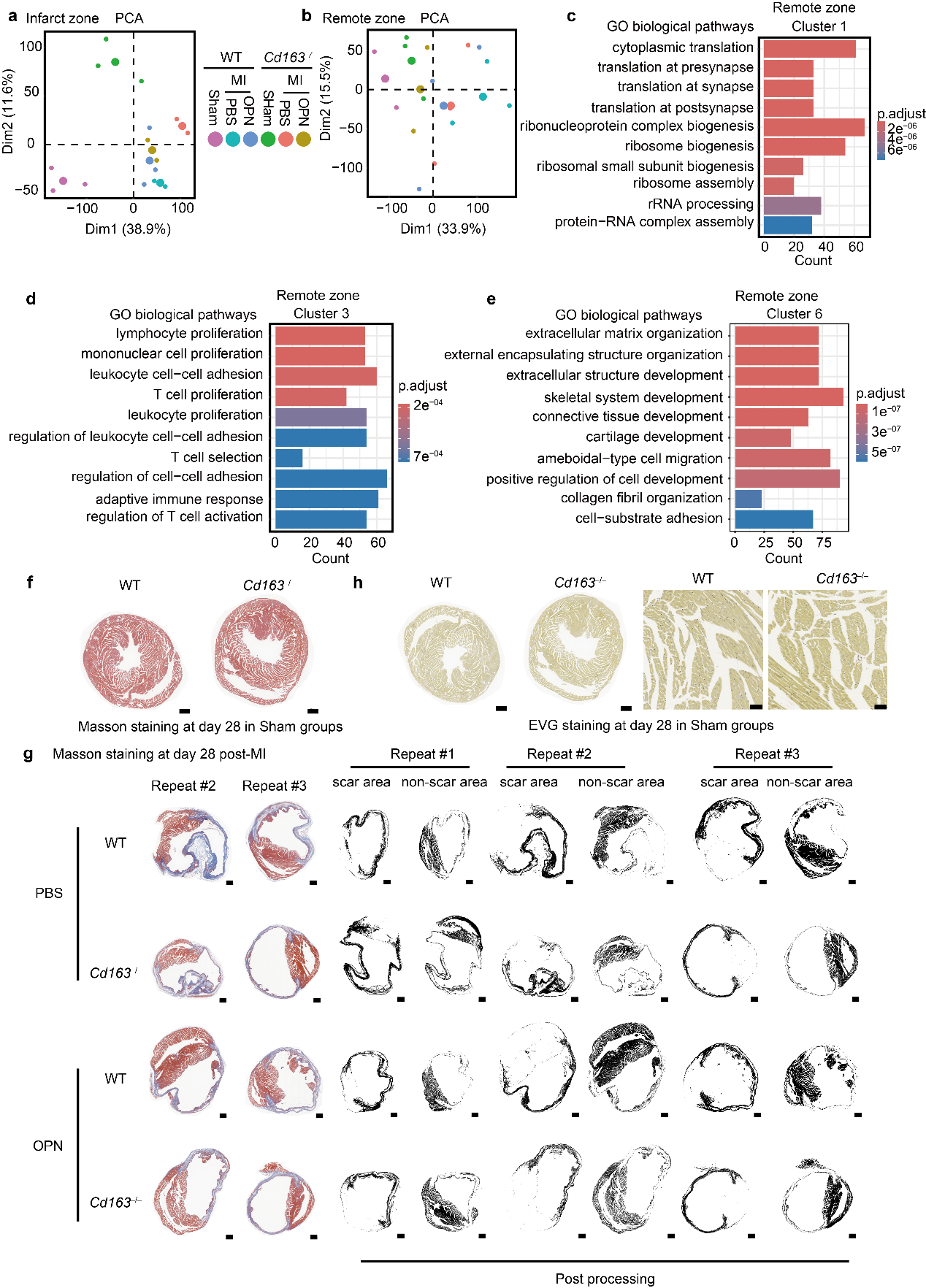
**

**Figure S10. OPN administration improved cardiac repair in CD163-deficient mice following MI. (a-c)** RNA-seq of infarct and remote zones at day 28 post-MI. Mice received 200 ng OPN or PBS injected into the infarct zone during MI or underwent sham surgery (WT and *Cd163*^−^*^/^*^−^). **(a)** PCA of genes in the infarct zone across groups. Sample sizes: n = 3, 3, 3, 3, 2, 2, with each sample pooled from 2–3 mice. **(b)** PCA of genes in the remote zone across groups. n = 3, 3, 3, 3, 3, 2, with each sample pooled from 2–3 mice. GO biological pathway enrichment analysis of **(c)** cluster 1, **(d)** cluster 3 and **(e)** cluster 6 genes identified in Fig. 6b. **(f)** Masson’s trichrome staining of heart sections from sham groups at day 28 post-surgery. Scale bars: 500 µm **(g)** Masson’s trichrome staining of heart sections from the indicated groups. Processed images highlighting scar and non-scar areas are shown. Scale bars: 500 µm. **(h)** EVG staining of heart sections from sham groups at day 28 post-surgery. Scale bars: 500 µm and 50 µm.


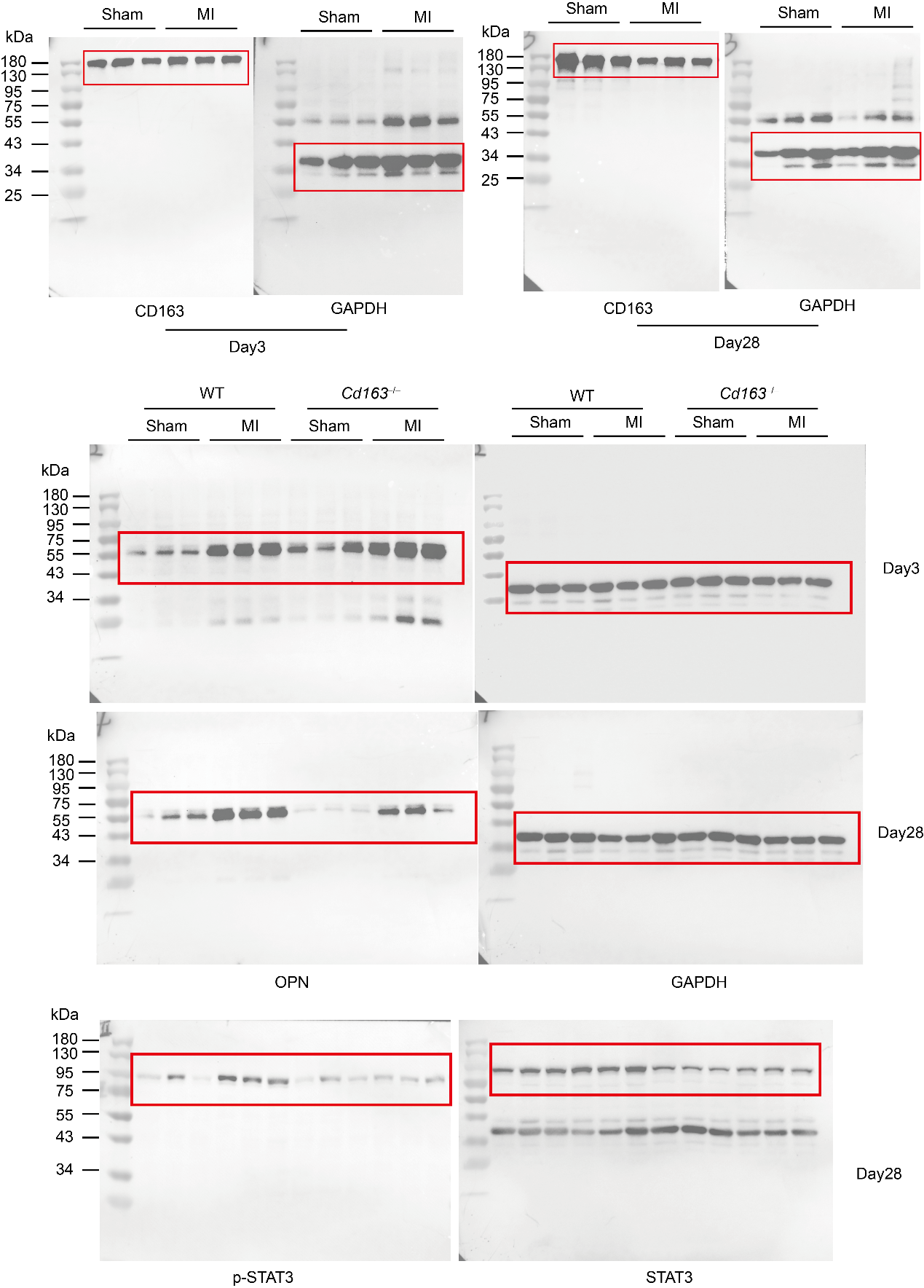


**Figure S11. Whole gel images of western blot.**

Supplementary Tables

**Table S1. Key resource table**

| **Reagent or resource** | **Identifier** | | **Source** | |
| --- | --- | --- | --- | --- |
| **Antibodies** |  | |  | |
| PE anti-mouse CD163 Antibody | 156703 | | Biolegend | |
| PE anti-mouse CD45 Antibody | 103105 | | Biolegend | |
| Brilliant Violet 650 anti-mouse CD192 (CCR2) Antibody | 150613 | | Biolegend | |
| PE/Cyanine7 anti-mouse Tim-4 Antibody | 130009 | | Biolegend | |
| Brilliant Violet 510 anti-mouse I-A/I-E Antibody | 107635 | | Biolegend | |
| PerCP/Cyanine5.5 anti-mouse/human CD11b Antibody | 101227 | | Biolegend | |
| Brilliant Violet 421 anti-mouse F4/80 Antibody | 123131 | | Biolegend | |
| Brilliant Violet 605 anti-mouse CD45 Antibody | 103139 | | Biolegend | |
| CD163 Rabbit mAb for Western blot | A25206 | | abclonal | |
| Anti-CD163 Rabbit pAb for IHC | GB113751 | | Servicebio | |
| Osteopontin Mouse mAb | A21084 | | abclonal | |
| GAPDH Rabbit mAb | A19056 | | abclonal | |
| STAT3 Rabbit mAb | A19566 | | abclonal | |
| Phospho-STAT3-Y705 Rabbit mAb | AP0705 | | abclonal | |
| HRP-conjugated Goat anti-Mouse IgG (H+L) | AS003 | | abclonal | |
| HRP-conjugated Goat anti-Rabbit IgG (H+L) | AS014 | | abclonal | |
| **Chemicals, kit and buffer** |  | |  | |
| Human CD163 DuoSet ELISA | DY1607-05 | | R&D | |
| 2,3,5,Triphenyl-2H-Tetrazolium   Chloride | 0765-1 | | lablead | |
| Isoflurane | R510 | | RWD | |
| Recombinant Mouse Osteopontin/SPP-1 Protein | RP02806 | | abclonal | |
| Recombinant Mouse CD163 Protein | RP02115 | | abclonal | |
| Mouse Serum Albumin | 36137ES01 | | yeasen | |
| MobiCube 5’ RNA-seq kit | MS060221 | | mobidrop | |
| Illumina® Stranded mRNA Prep Ligation | 20040534 | | Illumina | |
| Masson’s trichrome staining kit | G1006 | | Servicebio | |
| Verhoeff's Van Gieson Staining kit | GP1035 | | Servicebio | |
| Liberase | 5401127001 | | Roche | |
| Dnase I | D5025 | | Lablead | |
| Percoll | 17089101 | | Cytiva | |
| Prestained Protein marker | P1018 | | Lablead | |
| WesternBright ECL HRP substrate | K-12045-D50 | | Advansta | |
| M-PER Mammalian Protein Extraction Reagent | 78501 | | Thermo | |
| Pierce BCA Protein Assay Kit | 23225 | | Thermo | |
| cOmplete Protease Inhibitor Cocktail | 04693116001 | | Roche | |
| PhosSTOP | 4906845001 | | Roche | |
| FcR blocking reagent | 130-092-575 | | Miltenyibiotec | |
| Fixable Viability Dye eFluor 780 | 65-0865-14 | | Thermo Fisher | |
| RPMI 1640 | L210KJ | | Basalmedia Technologies | |
| Trizol | 15596026 | | Thermo Fisher | |
| **Software and Algorithms** |  | |  | |
| R (v4.2.1) |  | |  | |
| HISAT (v2.2.1) |  | |  | |
| Featurecounts (v2.0.1) |  | |  | |
| Limma v3.56.1 |  | |  | |
| Gseabase (v1.62.0) |  | |  | |
| Clusterprofiler (v4.8.1) |  | |  | |
| Trim-Galore (version 0.6.10) |  | |  | |
| MobiVision v3.2 |  | | mobidrop | |
| Seurat R package (v2.6) |  | |  | |
| Flow Jo (v.10.0.7) |  | | Becton Dickinson | |
| Prism 9 |  | | Graphpad | |
| Fiji (2.17.0) |  | |  | |
| SPSS (29.0) |  | | IBM | |
| **Mice** |  | |  | |
| C57BL6J mice |  | | Gempharmatech | |
| *Cd163*^−^*^/^*^−^ mice |  | | Ref 17, 18 | |
| **Primers** |  | |  | |
| Su (Forward) | GTGTTCCAAAGTGGGAGGAG | | *Cd163* deficiency allele | |
| Laz (Reverse) | | GTCTGTCCTAGCTTCCTCACTG | | *Cd163* deficiency allele |
| TDF (Forward) | TCATTCCAGGAGAAGTGCCC | | WT allele | |
| TDR (Reverse) | CCAAGGCAAATCCCTCTCAG | | WT allele | |

**Table S2. Characteristics of subjects**

|  | **Control** | **n** | **Heart failure** | **n** | ***P*-value** |
| --- | --- | --- | --- | --- | --- |
| Age, year | 66.0 (60.0 - 72.5) | 60 | 71.0 (64.8 - 77.0) | 30 | 0.075 |
| Male, n (%) | 36 (60.0%) | 60 | 20 (66.7%) | 30 | 0.647 |
| Female, n (%) | 24 (40.0%) | 60 | 40（33.3%） | 30 | 0.647 |
| Body mass index, kg/m^2^ | 26.2 (24.0 - 28.6) | 60 | 24.7 (21.4 - 27.2) | 29 | 0.095 |
| Smoking history, n (%) | 24 (40.0%) | 60 | 12 (40.0%) | 30 | 1.000 |
| Alcohol history, n (%) | 16 (26.7%) | 60 | 4 (13.3%) | 30 | 0.186 |
| Systolic blood pressure, mm Hg | 142.5 (130.0 - 158.8) | 60 | 133.5 (115.8 - 150.5) | 30 | 0.043* |
| Diastolic blood pressure, mm Hg | 84.5 (70.5 - 89.8) | 60 | 75.0 (66.5 - 94.3) | 30 | 0.722 |
| **Echocardiographic findings** |  |  |  |  |  |
| Left ventricle ejection fraction, % | 65.5 (60.0 - 70.0) | 60 | 35.5 (31.8 - 44.0) | 30 | <0.001*** |
| Left atrial diameter, mm | 38.0 (34.0 - 42.8) | 60 | 42.0 (39.0 - 49.0) | 30 | 0.003** |
| Interventricular septal thickness at end-diastole, mm | 10.8 (9.5 - 12.6) | 60 | 9.3 (8.7 - 10.1) | 30 | <0.001*** |
| Left ventricular end diastolic diameter, mm | 46.0 (43.0 - 51.0) | 60 | 60.5 (55.5 - 65.0) | 30 | <0.001*** |
| Left ventricular end systolic diameter, mm | 29.0 (27.0 - 34.0) | 60 | 50.5 (41.5 - 56.0) | 30 | <0.001*** |
| left ventricular posterior wall thickness at end-diastole, mm | 10.0 (9.0 - 11.3) | 60 | 9.2 (8.0 - 10.0) | 30 | 0.059 |
| **Laboratory findings** |  | 60 |  |  |  |
| N-terminal pro b-type natriuretic peptide, ng/L | 102.0 (29.9 - 745.0) | 59 | 3043.8 (835.5 - 6450.1) | 30 | <0.001*** |
| High-sensitive cardiac troponin T, ng/L | 16.0 (7.5 - 32.4) | 59 | 40.3 (19.9 - 66.5) | 30 | <0.001*** |
| High-sensitive C-Reactive Protein, mg/L | 2.3 (0.9 - 8.4) | 54 | 6.0 (2.2 - 13.3) | 30 | 0.02* |
| Serum creatinine, μmol/L | 80.7 (62.5 - 105.7) | 60 | 102.5 (82.0 - 145.8) | 30 | 0.005** |
| Estimated glomerular filtration rate, mL/min/1.73m^2^ | 76.0 (58.8 - 93.0) | 60 | 58.8 (43.8 - 77.5) | 30 | 0.004** |
| Triglycerides, mmol/L | 1.7 (1.2 - 3.4) | 60 | 1.3 (0.9 - 2.0) | 30 | 0.023* |
| Cholesterol, mmol/L | 4.5 (3.6 - 6.0) | 60 | 4.0 (3.4 - 4.5) | 30 | 0.093 |
| High-density lipoprotein cholesterol, mmol/L | 1.1 (0.9 - 1.4) | 60 | 1.0 (0.9 - 1.2) | 30 | 0.171 |
| Low-density lipoprotein cholesterol, mmol/L | 2.6 (2.1 - 3.6) | 60 | 2.5 (1.9 - 2.9) | 30 | 0.302 |
| **Comorbidities, n (%)** |  |  |  |  |  |
| Diabetes mellitus | 17 (28.3%) | 60 | 16 (53.3%) | 30 | 0.036* |
| Dyslipidemia | 29 (48.3%) | 60 | 10 (33.3%) | 30 | 0.259 |
| Chronic kidney disease | 10 (16.7%) | 60 | 16 (53.3%) | 30 | <0.001*** |
| Atrial fibrillation | 11 (18.3%) | 60 | 4 (13.3%) | 30 | 0.765 |
| Cerebrovascular accident | 4 (6.7%) | 60 | 5 (16.7%) | 30 | 0.154 |
| **Medicines, n (%)** |  |  |  | 30 |  |
| Loop diuretics | 9 (15.0%) | 60 | 24 (80.0%) | 30 | <0.001*** |
| Mineralocorticoid receptor antagonists | 5 (8.3%) | 60 | 21 (70.0%) | 30 | <0.001*** |
| Sodium–glucose cotransporter 2 inhibitors | 20 (33.3%) | 60 | 22 (73.3%) | 30 | <0.001*** |
| Inotropes | 0 (0%) | 60 | 1 (1.1%) | 30 | 0.333 |
| Vericiguat | 0 (0%) | 60 | 11 (36.7%) | 30 | <0.001*** |
| Statins | 57 (95.0%) | 60 | 28 (93.3%) | 30 | 1.000 |
| Angiotensin II receptor blockers | 17 (28.3%) | 60 | 16 (53.3%) | 30 | 0.036* |
| Angiotensin converting enzyme inhibitors | 7 (11.7%) | 60 | 0 (0%) | 30 | 0.090 |
| Beta blockers | 39 (65.0%) | 60 | 27 (90.0%) | 30 | 0.012* |
| Proprotein convertase subtilisin/kexin type 9 inhibitors | 7 (11.7%) | 60 | 5 (16.7%) | 30 | 0.525 |
| Oral antidiabetic drugs | 14 (23.3%) | 60 | 10 (33.3%) | 30 | 0.324 |
| Thrombin inhibitors | 9 (15.0%) | 60 | 10 (33.3%) | 30 | 0.057 |
| Antiplatelet drug | 32 (53.3%) | 60 | 26 (86.7%) | 30 | 0.002** |
| Calcium channel blockers | 31 (51.7%) | 60 | 5 (16.7%) | 30 | 0.001** |

Data are presented as medians with interquartile ranges (Q1–Q3) for non-normally distributed variables, with P values calculated using the Mann–Whitney test; or as counts with percentages for categorical variables, with P values calculated using the chi-square test. *P*-values are denoted as follows: *, *p* < 0.05; **, *p* < 0.01; and ***, *p* < 0.001.
